# Proteomic differences in recombinant CHO cells producing two similar antibody fragments

**DOI:** 10.1002/bit.25957

**Published:** 2016-03-16

**Authors:** Wolfgang Sommeregger, Patrick Mayrhofer, Willibald Steinfellner, David Reinhart, Michael Henry, Martin Clynes, Paula Meleady, Renate Kunert

**Affiliations:** ^1^ Vienna Institute of BioTechnology (VIBT)University of Natural Resources and Life SciencesMuthgasse 18, B, 5th Floor1190 ViennaAustria; ^2^ Polymun Scientific GmbHKlosterneuburgAustria; ^3^ Bilfinger Industrietechnik Salzburg GmbHSalzburgAustria; ^4^ National Institute for Cellular Biotechnology (NICB)Dublin City UniversityDublin 9Ireland

**Keywords:** Chinese hamster ovary cells, bottleneck, endoplasmic reticulum, secretion, specific productivity

## Abstract

Chinese hamster ovary (CHO) cells are the most commonly used mammalian hosts for the production of biopharmaceuticals. To overcome unfavorable features of CHO cells, a lot of effort is put into cell engineering to improve phenotype. “Omics” studies investigating elevated growth rate and specific productivities as well as extracellular stimulus have already revealed many interesting engineering targets. However, it remains largely unknown how physicochemical properties of the recombinant product itself influence the host cell. In this study, we used quantitative label‐free LC‐MS proteomic analyses to investigate product‐specific proteome differences in CHO cells producing two similar antibody fragments. We established recombinant CHO cells producing the two antibodies, 3D6 and 2F5, both as single‐chain Fv‐Fc homodimeric antibody fragments (scFv‐Fc). We applied three different vector strategies for transgene delivery (i.e., plasmid, bacterial artificial chromosome, recombinase‐mediated cassette exchange), selected two best performing clones from transgene variants and transgene delivery methods and investigated three consecutively passaged cell samples by label‐free proteomic analysis. LC‐MS‐MS profiles were compared in several sample combinations to gain insights into different aspects of proteomic changes caused by overexpression of two different heterologous proteins. This study suggests that not only the levels of specific product secretion but the product itself has a large impact on the proteome of the cell. Biotechnol. Bioeng. 2016;113: 1902–1912. © 2016 The Authors. *Biotechnology and Bioengineering* Published by Wiley Periodicals, Inc.

## Introduction

Chinese hamster ovary (CHO) cells are the most frequently used mammalian host for the production of biopharmaceuticals (Walsh, 2014). For a better understanding of the biological background and to improve the production system “omics”‐based research has given insights into cellular changes triggered by overexpression, higher growth rates or extracellular stimulus like hypothermic cultivation, butyrate treatment, or hyperosmotic conditions (Datta et al., 2013; Kildegaard et al., 2013; Kim et al., 2012). We hypothesize that the proteome of production clones is additionally and considerably influenced by the nascent and mature recombinant protein, characterized by distinct biochemical features (e.g., structure, stability, surface charge distribution) and challenging post‐translational modifications as well as secretion. To create genetically more defined and comparable recombinant cell lines, the CHO DUKXB11‐RMCE host cell line capable of targeted gene integration by recombinase‐mediated cassette exchange (RMCE) was described (Mayrhofer et al., 2014) and used for recombinant protein expression of scFv‐Fc versions of the two anti‐HIV‐1 antibodies 3D6 and 2F5 (Kunert et al., 1998) under isogenic conditions. Although, the same RMCE host cell line was used and transgene copy numbers as well as levels of transcript were identical, a twofold difference in specific productivity between 2F5‐ and 3D6‐scFv‐Fc producers was observed. Following this observation, we applied two additional strategies of transgene delivery and established 2F5‐ and 3D6‐scFv‐Fc producers, once with a common plasmid strategy and secondly with the *Rosa26* bacterial artificial chromosome (BAC) strategy (Blaas et al., 2009, 2012; Kunert and Casanova, 2013; Mader et al., 2013; Zboray et al., 2015), applying the same CHO DUKXB11 host cell line that was used for the generation of the RMCE cell line. Independent from the transgene delivery system, we regularly identified clones with higher specific productivities for 3D6‐scFv‐Fc compared to 2F5‐scFv‐Fc producing cell lines. These findings support the assumption that the protein's physicochemical properties in particular the unique variable heavy domain (*V*_H_)/variable light domain (*V*_L_) sequences of the antibody fragment, trigger different intracellular responses within the recombinant cells.

In this study, we aimed to identify differences on a proteomic level in recombinant CHO cells producing the scFv‐Fc version of either 2F5 or 3D6 rather than comparing the recombinant cell lines to the respective host cell line. In order to minimize bias caused by clonal selection, screening, and the genomic variability of CHO cells, we included the two best producing clones for each scFv‐Fc variant of three transgene delivery methods (RMCE, plasmid, and BAC). Understandably, not only the transgene but also the different vector systems generated cell lines with varying specific production rates for both products. Samples from the 12 clones (two best producers, two products, three transgene delivery methods) were collected from three consecutive passages and unfractionated whole‐cell‐lysates were analyzed using quantitative label‐free LC‐MS proteomic analyses and bioinformatics tools. Evaluation of LC‐MS data was performed by two types of comparisons. The “transgene comparison” was applied to identify proteomic changes caused by the two products while the “transgene delivery comparison” aimed to elucidate proteomic changes resulting from elevated production rates. In summary, we could show that not only the specific product secretion rate but also the recombinantly produced protein itself considerably influences the cell's physiology. These findings suggest that host engineering strategies might only work for a subset of recombinant proteins.

## Materials and Methods

### Antibody Fragments

3D6‐Fv (PDB: 1DFB) and 2F5‐Fv (PDB: 2F5B), respectively, were combined via a (GGGGS)_3_ linker to the human immunoglobulin G (IgG)‐1 Fc region (GenBank, CAA49866). 2F5‐scFv‐Fc and 3D6‐scFv‐Fc sequence alignment (79.3% identity) is shown in Supplemental Figure S1. Nucleotide sequences were codon optimized and synthesized (Geneart, Regensburg, Germany).

### Cell Culture

3D6‐ and 2F5‐scFv‐Fc expressing cell lines were used for the generation of samples for proteomic and thermal stability analyses. Both antibody fragments were produced using three different vector systems each transfected in the same protein‐free adapted CHO‐DUKX‐B11 (ATCC CRL‐9096) (Urlaub and Chasin, 1980) host cell lines. The applied transgene delivery systems were indicated by (i) common plasmid vectors; (ii) *Rosa26* BACs; and (iii) RMCE, for the generation of the recombinant cell lines. The two best performing clones for each strategy and product were used in this study (*n* = 12). The cell line establishment of the 12 clones has been previously described (Mader et al., 2013; Mayrhofer et al., 2014). All clones were cultivated in suspension in 125‐mL spinner flasks (Techne, Thermo Fisher Scientific, Waltham, MA) at 37°C, 7% CO_2_, and 50 rpm. ProCHO5 (Lonza, Basel, Switzerland) was used for all clones supplemented with 4 mM l‐glutamine and hypoxanthine/thymidine (HT) without any selection pressure. Cells were passaged into fresh media every 3–4 days.

### Cell Counting and Viability Determination

The cell concentration was calculated by counting the nuclei of cells lyzed in 0.1 M citric acid and 2% (w/w) Triton X 100 with the particle counter Z2 (Beckman Coulter, Brea, CA). Viability was determined by trypan blue exclusion method using a Neubauer cell counting chamber. Growth rate *μ* (d^−1^) was calculated according to Equation (1) where X (cells) represents the total number of viable cells and *t* (d) the cultivation time in days.

(1) $$\mu =\ln\;\left(\frac{X_{1}}{X_{0}}\right)\times \frac{1}{\left(t_{1}-t_{0}\right)}$$

### Product Concentration and Specific Productivity

The product concentration in the cell supernatants was determined by sandwich enzyme‐linked immunosorbent assay (ELISA) as previously described (Mader et al., 2013; Mayrhofer et al., 2014). Briefly, 96‐well Maxisorp plates (Nunc, Thermo Fisher Scientific) were coated with polyclonal goat anti‐human IgG (γ‐chain specific) (I3382, Sigma–Aldrich, St. Louis, MO). Goat anti‐human IgG horseradish peroxidase conjugate (γ‐chain specific) (62‐8420, Life Technologies, Carlsbad, CA) was used as the detection antibody. Staining was initiated with orthophenylediamine and H_2_O_2_ and the resulting color reaction was measured at 492 nm and a reference wavelength of 620 nm on a micro‐plate reader (Tecan, Männedorf, Switzerland). Specific productivity qP (pg × cell^−1^ × day^−1^) was calculated according to Equation (2) where *P* (pg) represents the product amount.

(2) $$qP=\mu \times \frac{\left(P_{1}-P_{0}\right)}{\left(X_{1}-X_{0}\right)}$$

### Differential Scanning Calorimetry

2F5 and 3D6 antibody fragments were purified by Protein A chromatography from pooled culture supernatants from all clones (*n* = 12) using a 1 mL pre‐packed MabSelect SuRe resin on an ÄKTA Purifier (both GE Healthcare, Little Chalfont, UK) according to the manufacturer's instructions. Eluates were desalted and buffer‐exchanged to 40 mM phosphate, 150 mM NaCl, pH 6.0 using PD MidiTrap G‐25 units (GE Healthcare) according to the manufacturer's instructions. Finally, sample quantification was performed on a NanoDrop spectrophotometer (Thermo Fisher Scientific) by applying the scFv‐Fc respective theoretical extinction coefficients. Thermal denaturation of the scFv‐Fc samples was monitored using automated differential scanning calorimetry (DSC). All DSC measurements were performed in duplicates on a VP‐DSC MicroCal LLC equipment (GE Healthcare). Protein solutions were sampled from 96‐well plates using the robotic attachment. The protein concentration of all samples was 2–3 µM. The temperature profile was recorded between 20 and 100°C with a scan rate of 1°C/min. The results were evaluated and fitted with the Origin 7.0 software (OriginLab, Northampton, MA). The unfolding states of the antibodies were fit using the non‐two state unfolding model within the software.

### Sample Preparation for Label Free LC‐MS Analysis

Sampling of each clone was performed at three consecutive passages (biological replicates). Ten million cells were harvested before each splitting by centrifugation and washed twice with cold PBS. Washed cell pellets were immediately snap frozen in liquid nitrogen and stored at −80°C prior to further preparation. Cells were thawed on ice and lyzed in lysis buffer (7 M urea, 2 M thiourea, 4% CHAPS, 30 mM Tris, pH 8.5) for 1 h at room temperature. Whole cell lysates were cleaned up using ReadyPrep 2D Cleanup Kit (Bio‐Rad Laboratories, Hercules, CA) and purified proteins resuspended in label free buffer (6 M urea, 2 M thiourea, 10 mM Tris, pH 8.0). Protein concentrations were determined in triplicate using a Bradford 1× Dye Reagent and a Quick start BSA standard (both Bio‐Rad). A total of 14 μg of protein were re‐suspended in 46.6 μL of 50 mM ammonium bicarbonate. Reduction was performed by adding 0.5 μL of 0.5 M DTT at 56°C for 20 min. Afterwards, samples were alkylated by adding 1.4 μL of 0.55 M iodoacetamide and then incubated for 35 min at room temperature. Digestion was performed by adding 1 μg MS grade Trypsin Gold (Promega, Fitchburg, WI) and 0.01% ProteaseMax (Promega) overnight at 37°C. Trifluoroacetic acid (TFA) was added to a final concentration of 0.5% to inactivate the trypsin. Samples were frozen at −20°C prior to analysis by LC‐MS/MS.

### Quantitative Label Free LC‐MS Analysis

Nano LC–MS‐MS was carried out essentially as previously described (Linge et al., 2014). Briefly, the analysis was performed using an Ultimate 3000 RSLCnano system (Dionex, Thermo Fisher Scientific) coupled to a hybrid linear ion trap/Orbitrap mass spectrometer (LTQ Orbitrap XL; Thermo Fisher Scientific). Samples were thawed and sonicated to ensure an even suspension and 1 μg of digested proteins were loaded onto a C18 trap column (C18 PepMap, 300 µm i.d. × 5 mm, 5 µm particle size, 100 µm pore size; Dionex) and desalted for 5 min. The trap column was then switched online with the analytical column (PepMap C18, 75 µm i.d. × 500 mm, 3 µm particle, and 100 µm pore size; Dionex), and peptides were eluted in a 300 min gradient. Data were acquired with Xcalibur software, version 2.0.7 (Thermo Fisher Scientific). The mass spectrometer was operated in data‐dependent mode and externally calibrated.

### Data Analysis

Differential proteomic analysis using label‐free LC‐MS/MS was carried out using Progenesis QI for proteomics version 1.0 (NonLinear Dynamics Limited, Newcastle upon Tyne, UK), essentially as recommended by the manufacturer and as previously described (Clarke et al., 2012). The raw data obtained from each of the LC‐MS/MS runs per sample was processed using Progenesis QI for proteomics software. Several criteria were used to filter the data before exporting the Progenesis output files to Proteome Discoverer 1.4 (Thermo Fisher Scientific) for protein identification: peptide features with adjusted ANOVA *P*‐value ≤0.05 between experimental groups, mass features with charge states from +1 to +3, and the number of isotopes was set to 3 or less. All MS/MS spectra were exported from Progenesis software as an mgf file and searched against CHO‐specific protein sequence databases, using a combination of the translated NCBI genomic database (Baycin‐Hizal et al., 2012) containing 24,927 entries (fasta file downloaded January 2014) and the expressed cDNA database (BB‐CHO) (Meleady et al., 2012) containing 14,627 entries, through Proteome Discoverer 1.4 and the search algorithms Mascot and SequestHT. The search parameters used for all searches on Proteome Discoverer 1.4 were as follows: precursor mass tolerance set to 20 ppm, fragment mass tolerance set to 0.6 Da; up to two missed cleavages were allowed, carbamidomethylation set as a fixed modification, and methionine oxidation set as a variable modification. For re‐importation back into Progenesis LC‐MS software for further analysis only peptide identifications with MASCOT ion peptide scores above 40 or peptides with XCorr scores >1.9 for singly charged ions, >2.2 for doubly charged ions and >3.75 for triply charged ions from Sequest analysis were accepted. To filter out target peptide spectrum matches (target‐PSMs) over the decoy‐PSMs, a fixed false discovery rate (FDR) of 1% was set at the peptide level. Proteins were only then considered as being differentially expressed between experimental groups if they had an adjusted ANOVA *P*‐value ≤0.05 and were identified by ≥2 peptides.

### Experimental Design

We used two different strategies to explore differences within the proteome of 2F5‐ and 3D6‐scFv‐Fc producers.

#### Transgene Comparison

**Figure 1:**
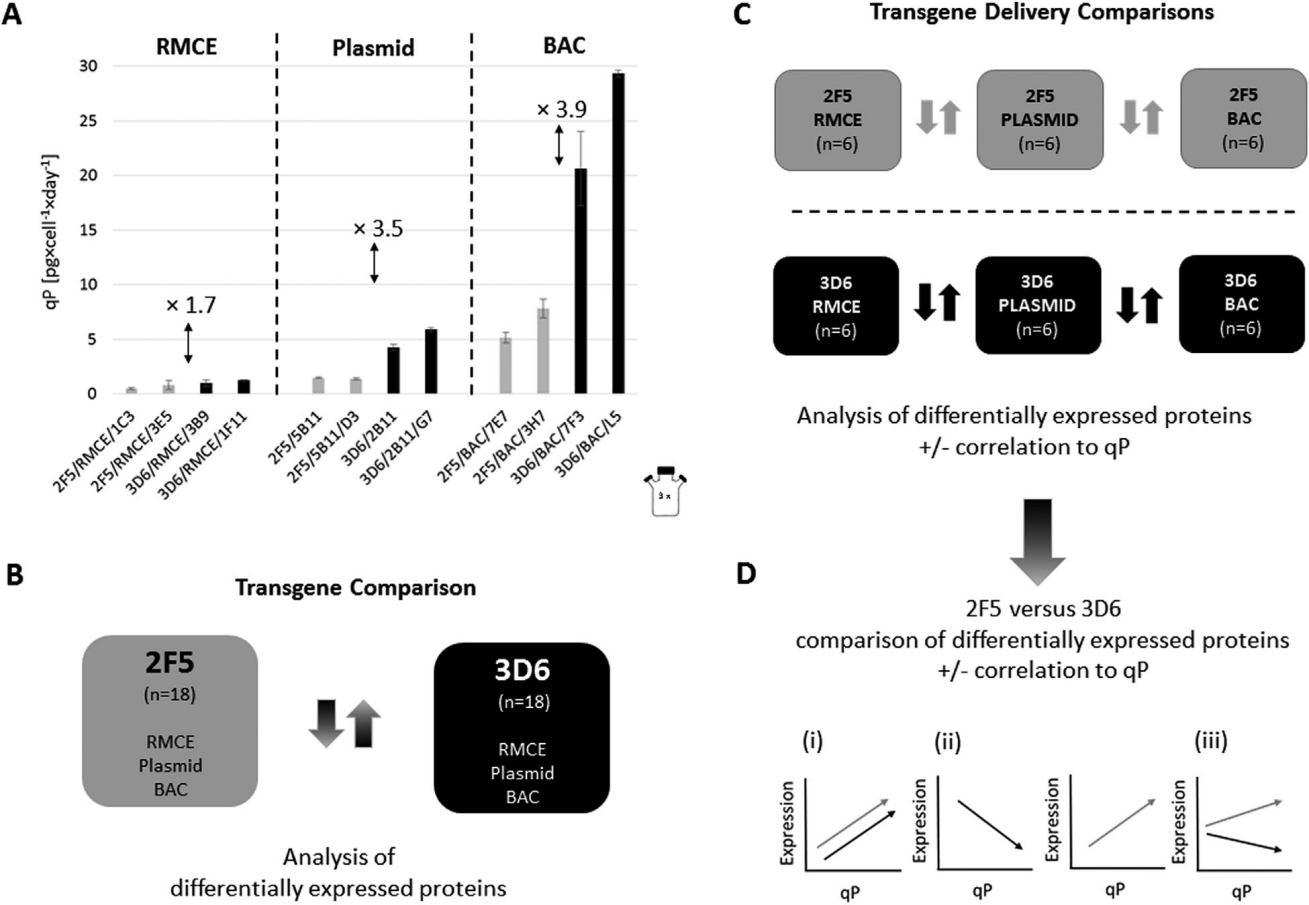
Schematic work‐flow representation: (**A**) Cell specific productivities were identified and samples for proteomic analysis were taken. (**B**) LC‐MS‐MS data of all 2F5‐scFv‐Fc producers were compared with 3D6‐scFv‐Fc data to explore global transgene‐specific proteome changes. (**C**) 2F5‐ and 3D6‐scFv‐Fc transgene delivery comparisons were performed and differentially expressed proteins with positive or negative correlation to qP were evaluated. (**D**) Subsequently, differentially expressed proteins that showed a ± correlation to qP in the in‐group comparisons of 2F5‐ and 3D6‐scFv‐Fc producers were compared to each other.

In a first approach, all 2F5‐scFv‐Fc samples (*n* = 18) were compared to all 3D6‐scFv‐Fc samples (*n* = 18) and differentially expressed proteins were evaluated (Fig. 1B). Using this transgene comparison, we could identify proteome changes which are related to the expressed product.

#### Transgene Delivery Comparison

In the second data evaluation approach, we performed 2F5‐ and 3D6‐scFv‐Fc transgene delivery comparisons of RMCE versus plasmid versus BAC (each *n* = 6, resulting from two clones analyzed in three consecutive passages) (Fig. 1C). Proteins that were statistically significant differentially expressed between RMCE, plasmid, and BAC clones were obtained and only expression profiles that positively or negatively correlated to the specific productivity of the distinct products caused by the use of different transgene delivery strategies were taken into account and used for further comparison of 2F5‐scFv‐Fc and 3D6‐scFv‐Fc differential expression profiles (Fig. 1D). This strategy allowed us to identify proteins that are up/down regulated with higher specific productivities in both groups. Furthermore, we could identify proteins that were differentially expressed only in one of the groups as well as protein expression profiles that were opposing between the two groups.

### Pathway Analysis

Identified differential proteins were manually assigned to mouse official gene IDs, according to the protein names, for further gene list analysis using GeneCodis3 (http://genecodis.cnb.csic.es/) (Carmona‐Saez et al., 2007; Nogales‐Cadenas et al., 2009; Tabas‐Madrid et al., 2012).

### Western Blot

Cellular protein lysates of relevant cell clones were pooled to confirm the experimental data of the proteomics comparisons. A total of 15 μg of total protein was mixed with 2× Laemmli sample buffer, heated to 95°C for 5 min and cooled prior to loading on 4–12% NuPAGE Bis‐tris pre‐cast gels (Life Technologies). Resolved proteins were electrophoretically blotted to Amersham Hybond P membranes (GE Healthcare, Buckinghamshire, UK). Membranes were blocked in 5% milk powder dissolved in 0.1% Tween in Tris‐Buffered Saline (TBST), washed with TBST, and incubated with primary antibody (rabbit polyclonal antibody (pAb) to heat shock protein 60 (Hspd1), ab46798; rabbit pAb to catalase (Cat), ab1877; rabbit pAb to SERPINB1, ab47731, Abcam, Cambridge, UK) diluted in TBST including 5% milk powder. Subsequently, membranes were washed and incubated with secondary HRP‐conjugated antibodies (Goat anti‐rabbit pAb, P0448 Dako, Agilent Technologies, Santa Clara, CA) diluted in TBST including 5% milk powder. Western blots were developed using WesternBright ECL Spray (Advansta, Menlo Park, CA). Subsequently, the membranes were washed in TBST and re‐probed with loading control antibodies (mouse mAb to ß‐actin (Actb), ab8226, Abcam or mouse mAb to α‐tubulin (Tuba1a), Sigma–Aldrich) and secondary antibody (goat anti‐mouse pAb, P0447, Dako) as described above.

## Results

### Cell Culture

For each product (2F5‐ and 3D6‐scFv‐Fc) and each strategy of transgene delivery (RMCE, plasmid, and BAC), the two best performing cell lines identified by specific productivity and growth rate were cultivated (*n* = 12). The recombinant scFv‐Fc producers were routinely cultivated in spinner flasks and samples for proteomic analysis were taken before passaging during three consecutive passages (biological replicates) at high viabilities (>90%). Cell line specific parameters were determined and are summarized in Table I and depicted in Figure 1A. For both products, the clones generated with RMCE had a low specific productivity, clones generated using plasmids displayed a medium qP and BAC clones displayed a high specific production rate. Overall, the specific productivities of the 3D6‐scFv‐Fc producers were always higher compared to the 2F5‐scFv‐Fc clones. The mean difference between 2F5‐ and 3D6‐scFv‐Fc clones was 1.7× (RMCE), 3.5× (plasmid), and 3.9× (BAC). Growth rate correlated slightly negatively with qP and was highest for the RMCE‐generated 2F5‐ and 3D6‐scFv‐Fc producers with only minor differences between them.

**Table I:** Cell line performances in terms of specific growth rates and specific productivities. Comparison of 3D6 scFv‐Fc clones and 2F5scFv‐Fc clones generated with different strategies for transgene delivery

Clone	*μ* (d^−1^)	Mean *μ*	qP (pg × cell^−1^ × d^−1^)	Mean qP	Fold diff. in qP
2F5/RMCE/1C3	0.47	0.46	0.48	0.66	1.70
2F5/RMCE/3E5	0.46		0.84		
3D6/RMCE/3B9	0.38	0.40	1.00	1.12	
3D6/RMCE/1F11	0.43		1.24		
2F5/PLASMID/5B11	0.40	0.39	1.49	1.45	3.50
2F5/PLASMID/5B11/D3	0.38		1.42		
3D6/PLASMID/2B11	0.35	0.36	4.25	5.08	
3D6/PLASMID/2B11/G7	0.38		5.91		
2F5/BAC/7E7	0.30	0.33	5.17	6.49	3.85
2F5/BAC/3H7	0.36		7.81		
3D6/BAC/7F3	0.29	0.35	20.62	24.97	
3D6/BAC/L5	0.41		29.32		

### 2F5‐and 3D6‐scFv‐Fc Thermal Stability

**Figure 2:**
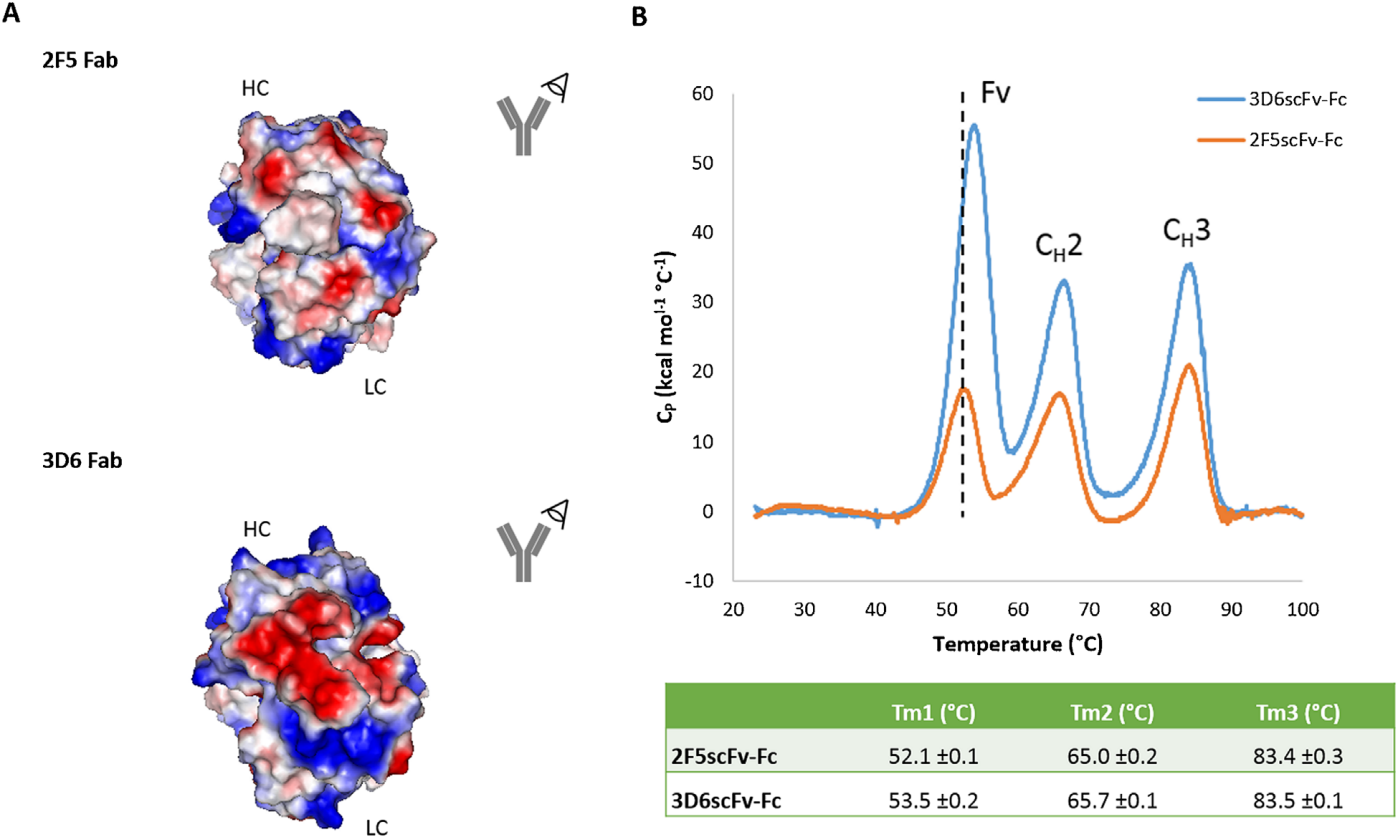
(**A**) Computationally modeled Fab fragment of the original 2F5 (PDB: 2F5B) and 3D6 (PDB: 1DFB) IgG using the PyMOL Molecular Graphics System, Version 1.3, Schrödinger, LLC. The surface model is colored by the underlying residue charge: red is negative, blue is positive, and white is neutral. (**B**) DSC thermogram of 2F5‐ and 3D6‐scFv‐Fc. The Fv, CH2, and CH3 domains and their respective unfolding transitions of are indicated.

A typical human IgG1 DSC curve contains three unfolding transitions of the constant heavy domains C_H_2, C_H_3 and fragment, antigen binding (Fab) domain. The herein tested single chain antibody fragments revealed three unfolding transitions that superimposed quite well (Fig. 2). However, some differences of the respective melting temperatures could be identified. Unfolding of the C_H_3 domain was almost identical for both 3D6‐ and 2F5‐scFv‐Fc at ∼83°C. The unfolding transition of the C_H_2 domain of 2F5‐scFv‐Fc (65.0 ± 0.2°C) occurred slightly earlier than that of 3D6scFv‐Fc (65.7 ± 0.1°C). The Fv's showed the lowest resistance to thermal unfolding and occurred 1.4°C earlier for 2F5‐scFv‐Fc (52.1 ± 0.1) than for 3D6‐scFv‐Fc (53.5 ± 0.2).

### Proteomics

We performed label‐free LC‐MS proteomic analyses of recombinant CHO cell lines producing two different types of scFv‐Fc fragments to explore changes caused by the recombinant protein (Fig. 1B) as well as differences evoked by elevated specific production rates (Fig. 1C and D) caused by the use of different transgene delivery strategies.

#### Transgene Comparison

To gain insight into proteomic changes caused by the type of recombinant scFv‐Fc being produced, we compared all 2F5‐ (*n* = 18) with all 3D6‐scFv‐Fc (*n* = 18) samples. Principal component analysis of all statistically significant differential peptides (*P* ≤ 0.05) shows a clear separation between 2F5‐ and 3D6‐scFv‐Fc samples (Supplemental Fig. S2). We could identify 60 proteins being differentially expressed between the two groups (*P* ≤ 0.05; ≥1.2‐fold change; ≥2 peptides used for quantification) (Supplemental Table SI). A selection of the identified differential expressed proteins is listed in Table II. Peptides were also searched against a human database. However, we could not identify the recombinant product itself to be statistically significantly different between the heterogeneous groups of 2F5‐ and 3D6‐scFv‐Fc producers, most probably due to the quantitative variability of intracellular product in the heterogeneous sample group.

**Table II:** Transgene comparison: table of selected proteins differential between 2F5 (*n* = 18) and 3D6 scFv‐Fc (*n* = 18) samples (*P* ≤ 0.05; ≥1.2× fold change; ≥2 peptides used for quantification) and sorted by fold change

Description	Gene ID	Fold change	Highest mean	Function
Galectin‐3	Lgals3	1.75	2F5	Anti‐apoptosis; mRNA processing; extracellular matrix organization
DNA replication licensing factor MCM5	Mcm5	1.58	2F5	DNA replication initiation
60 kDa Heat shock protein, mitochondrial^a^	Hspd1	1.50	2F5	Protein folding; apoptosis
N‐acetyltransferase 10	Nat10	1.49	2F5	Histone acetylation; metabolic process
Nucleolin	Ncl	1.43	2F5	Chromatin decondensation
10 kDa Heat shock protein, mitochondrial	Hspe1	1.43	2F5	Protein folding
Importin‐5	Ipo5	1.27	2F5	Protein import into nucleus
Metastasis‐associated protein MTA2	Mta2	1.22	2F5	Histone deacetylation, DNA packaging; apoptosis
Glutathione S‐transferase P 2	Gstp2	2.74	3D6	Metabolic process
Glutathione S‐transferase P 1	Gstp1	2.19	3D6	Apoptosis; metabolic process; regulation of stress‐activated MAPK cascade
Peroxiredoxin‐1	Prdx1	1.76	3D6	Response to oxidative stress; cell proliferation; regulation of stress‐activated MAPK cascade
Golgi‐associated plant pathogenesis‐related protein 1	Glipr2	1.64	3D6	Regulation of ERK1 and ERK2 cascade
Protein disulfide‐isomerase A3	Pdia3	1.58	3D6	Folding; apoptosis
Catalase^a^	Cat	1.52	3D6	Response to oxidative stress
Calreticulin	Calr	1.47	3D6	Protein folding; proliferation
SEC23‐interacting protein	Sec23Ip	1.38	3D6	Intracellular protein transport; golgi organization
Protein disulfide‐isomerase A4	Pdia4	1.33	3D6	Protein secretion; protein folding
Thioredoxin reductase 1, cytoplasmic	Txnrd1	1.29	3D6	Response to oxidative stress; proliferation
Glutathione S‐transferase Mu 6	Gstm6	1.28	3D6	Metabolic process
Eukaryotic translation initiation factor 5A‐1	Eif5a	1.28	3D6	Apoptosis; proliferation; translation
Eukaryotic initiation factor 4A‐I	Eif4a1	1.26	3D6	Translation
Glutathione S‐transferase omega‐1 isoform 1	Gsto1	1.26	3D6	Metabolic process
DnaJ homolog subfamily C member 7	Dnajc7	1.23	3D6	Protein folding
Protein disulfide‐isomerase	P4Hb	1.23	3D6	Protein folding
Endoplasmin	Hsp90b1	1.23	3D6	Protein folding; apoptosis
GrpE protein homolog 1, mitochondrial	Grpel1	1.21	3D6	Protein folding

^a^ Results validated via Western blotting (Fig. 3).

**Figure 3:**
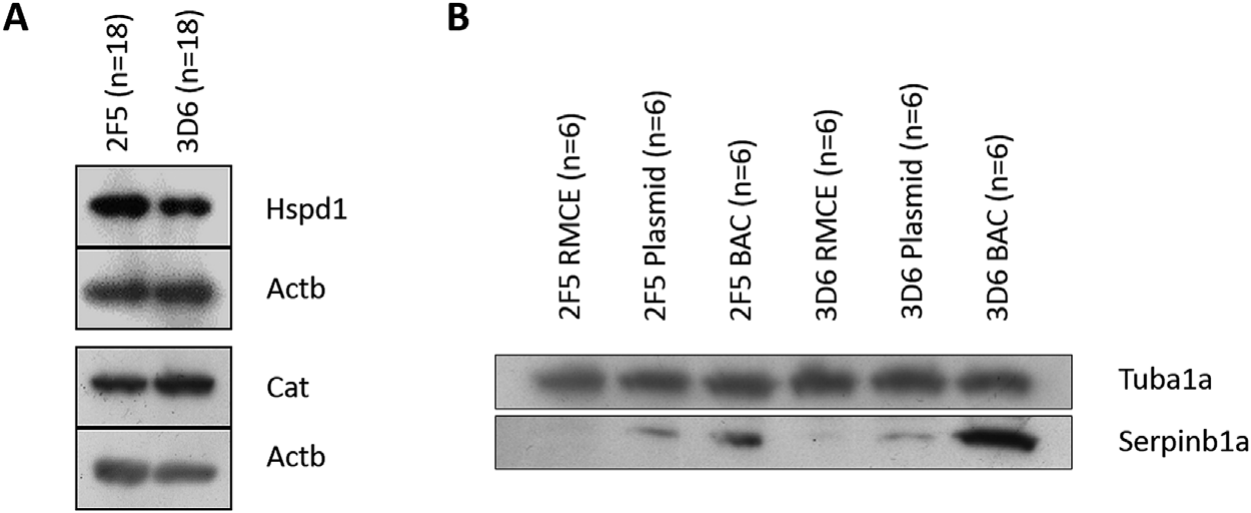
Western blot validations of proteomics results of the two differential (2F5s vs. 3D6s) proteins 60 kDa heat schock protein (Hspd1) and catalase showing increased expression of Hspd1 (fold difference: proteomic 1.50× and densitometric 1.24×) and decreased expression of catalase (fold difference: proteomic 1.52× and densitometric 1.36× in the group of 2F5s (*n* = 18) compared to 3D6s (*n* = 18) (**A**) and of the protein leukocyte elastase inhibitor A (Serpinb1a) showing increasing expression of Serpinb1a from RMCE to plasmid to BAC for 2F5‐scFv‐Fc and 3D6‐scFv‐Fc producers (**B**), where proteomics results showed a 5.8 and 18‐fold increase of Serpinb1a from RMCE to BAC for 2F5‐ and 3D6‐scFvFc producers, respectively; 15 μg of total protein from pooled lysates were loaded each and ß‐actin (Actb) or α‐tubulin (Tuba1a) was used as internal loading control.

Subsequently, we performed singular enrichment analysis using the official gene IDs (mouse equivalent) of the 60 identified proteins using the web‐based tool GeneCodis3. Singular enrichment analysis of GOSlim process revealed three biological processes to be statistically significant enriched (hypergeometric *P*‐value ≤0.05). These identified cellular processes were cell proliferation, protein folding, and extracellular matrix organization (Supplemental Table SVIII). Two of the identified differential proteins, the 60 kDa heat shock protein (Hspd1) and Catalase (Cat) were validated by western blots (Fig. 3A). The western blots of pooled lysates from all 2F5‐ (*n* = 18) and 3D6‐scFv‐Fc (*n* = 18) samples confirmed the overall higher abundance of Hspd1 in 2F5‐scFv‐Fc samples as well as higher abundance of Catalase in 3D6‐scFv‐Fc samples compared to ß‐actin (Actb) which was used as loading control.

#### Transgene Delivery Comparison

To explore proteomic changes caused by increasing product secretion rates resulting from different transgene delivery strategies for 2F5 and 3D6‐scFv‐Fc producing cell lines, we performed transgene delivery comparisons of RMCE (*n* = 6) versus plasmid (*n* = 6) versus BAC (*n* = 6) samples and evaluated the statistically significant proteins differentially expressed between RMCE, plasmid, and BAC clones. The outcome was firstly evaluated separately and afterwards combined. We identified 109 (for 2F5‐scFv‐Fc producers, Supplemental Table SII) and 212 (for 3D6‐scFv‐Fc producers, Supplemental Table SIII) differentially expressed proteins that displayed either a positive or negative correlation to qP or *μ* (highest relative abundance in BAC and lowest relative abundance in RMCE clones or vice versa; *P* ≤ 0.05; ≥1.5× fold change; ≥2 peptides used for quantification).

Subsequently, using this approach we were able to cluster the results into three groups of differentially expressed proteins:

##### (1) Expression pattern correlating to qP in both antibody groups (2F5 and 3D6)

**Figure 4:**
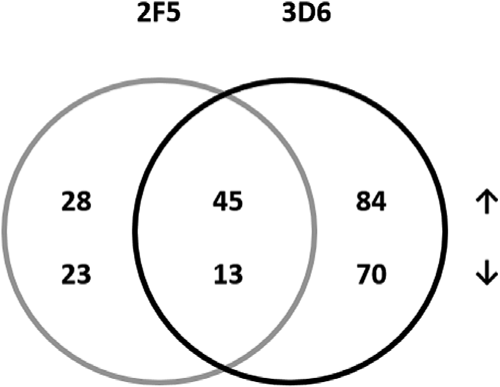
Venn diagram: Comparison of differentially expressed proteins correlating to qP identified in the 2F5‐ and 3D6‐scFv‐Fc transgene delivery comparisons; ↑: proteins positively correlating with qP, ↓: proteins negatively correlating with qP.

This first group of differentially expressed proteins was identified in 2F5‐ as well as in 3D6‐scFv‐Fc clones describing positive/negative correlation according to the transfection system—lowest relative abundance in the group of RMCE and highest relative abundance in the group of BAC generated clones or vice versa (reflects a correlation to qP or *μ*). We identified 61 proteins that show the same expression pattern in both group comparisons. Three proteins were poorly annotated hypothetical proteins and could not be assigned. Singular enrichment analysis of GOSlim process using the gene IDs of the 58 remaining proteins (Supplemental Table SIV, Fig. 4) revealed four processes to be statistically significant enriched (hypergeometric *P*‐value ≤0.05). These cellular processes were cell cycle, vesicle‐mediated transport, cell death, and transport in general (Supplemental Table SIX). Selected differentially expressed proteins showing the same expression pattern in both transgene delivery comparisons are listed in Table III. As internal control when searching the differential peptides against the human database, we detected the constant region of immunoglobulin (Ig) γ‐1 chain in both transgene delivery comparisons to be differential (Supplemental Fig. S3). 2F5 Ig γ‐1 chain was identified by 11 peptides with a 4.6‐fold change from RMCE to BAC. 3D6 Ig γ‐1 chain was identified by 7 peptides with a 10.1‐fold change from RMCE to BAC. To validate the proteomics results, we confirmed the differential expression of the protein leukocyte elastase inhibitor A (Serpinb1a) by Western blot (Fig. 3B). The increase of Serpinb1a levels from low in RMCE clones to high in BAC clones was confirmed for 2F5‐scFv‐Fc as well as in 3D6‐scFv‐Fc producers compared to α‐tubulin (Tub1a) which was used as loading control.

**Table III:** Transgene delivery RMCE (*n* = 6) versus plasmid (*n* = 6) versus BAC (*n* = 6); selected differential expressed proteins correlating with qP or *μ* identified for 2F5 scFv‐Fc and/or 3D6 scFv‐Fc producers (number of peptides used for quantitation ≥2; Anova *P*‐value ≤0.05; fold change ≥1.5×)

		3D6			2F5			
Description	Gene ID	Fold change	Max	Min	Fold change	Max	Min	Function
Leukocyte elastase inhibitor A^a^	Serpinb1a	18.04	BAC	RMCE	5.77	BAC	RMCE	Regulation of proteolysis
Ig γ‐1 chain C region	*IGHG1*	10.13	BAC	RMCE	4.62	BAC	RMCE	Recombinant product
Cathepsin B	Ctsb	5.14	BAC	RMCE	3.51	BAC	RMCE	Proteolysis
Galectin‐1	Lgals1	4.81	BAC	RMCE	4.91	BAC	RMCE	Proliferation
Gelsolin	Gsn	2.25	BAC	RMCE	2.57	BAC	RMCE	Vesicle‐mediated transport
Ras‐related protein Rab‐1A	Rab1	1.66	BAC	RMCE	2.01	BAC	RMCE	Vesicle‐mediated transport
Vesicle‐trafficking protein SEC22b	Sec22b	1.65	BAC	RMCE	1.69	BAC	RMCE	Vesicle‐mediated transport
Heme oxygenase 1	Hmox1	4.81	RMCE	BAC	3.41	RMCE	BAC	ER‐stress, proliferation
Proteasome‐associated protein ECM29‐like	Ecm29	3.71	RMCE	BAC	2.21	RMCE	BAC	Proteasome adaptor

^a^ Result validated via Western blotting (Fig. 3).

##### (2) Differentially expressed proteins identified in only one antibody group

The second group summarizes proteins that were only identified to be differential for one transgene delivery comparison (either 2F5‐ or 3D6‐scFv‐Fc). We identified 51 (2F5‐scFv‐Fc) and 154 (3D6‐scFv‐Fc) proteins that were only detected to be positively or negatively correlating to the specific productivities or growth rates in one of the two transgene delivery comparisons (RMCE vs. plasmid vs. BAC). However, many of these identified proteins were also identified in the respective other antibody transgene delivery comparison, but they were not correlating with the specific productivity or the growth rate (highest/lowest abundance in plasmid clones). These proteins are assumed to be not strictly regulated and therefore not further discussed. After exclusion of such proteins, we finally identified 9 and 32 proteins that were only detected to be differential in the transgene delivery comparison of the 2F5‐scFv‐Fc or 3D6‐scFv‐Fc, respectively, and not detected at all for the respective other group (Supplemental Tables SV and SVI).

##### (3) Differentially expressed proteins showing opposite expression patterns in the two antibody groups

We identified two proteins that showed exactly the opposite expression pattern in the two transgene delivery comparisons, positive correlation to qP in one transgene delivery comparison, and negative correlation in the other transgene delivery comparison (≥1.5‐fold change in at least one group). The identified proteins were Cullin‐associated NEDD8‐dissociated protein 1 (Cand1), a regulator of SCF ubiquitin ligases and Protein SON (Son), a mRNA splicing cofactor (Supplemental Table SVII). Cand1 and SON both were positively correlating with qP in the group of 3D6‐scFv‐Fc producers whereas negatively correlating with qP in the group of 2F5‐scFv‐Fc producers.

## Discussion

### Cell Culture

Three different strategies (RMCE, plasmid, and BAC) for transgene delivery were used to generate recombinant 2F5‐ and 3D6‐scFv‐Fc producers (Mader et al., 2013; Mayrhofer et al., 2014). The RMCE clones showed the lowest specific productivity and highest growth rate. Clones generated by *Rosa 26* BACs as transgene vehicles had the highest specific production rate and lowest growth rate for both products and clones generated by random integration of plasmid vectors were found in between (Fig. 1A). The RMCE clones have only one transgene copy integrated by recombination in a specific but undefined chromosomal locus. The plasmid clones, have presumably higher transgene copy numbers in unspecified loci. The clones that were generated using the *Rosa26* BAC technology have their transgenes in a well‐defined transcriptional highly active environment provided by the BAC but no fixed transgene copy number. This enables a simple explanation for the distinct specific production rates based on the transgene delivery method. Additionally, we identified significant differences between product‐specific expression rates of the two antibody fragments, which increased with increasing specific productivity. The mean qP difference between 2F5‐scFv‐Fc and 3D6‐scFv‐Fc was 1.7‐fold for the low‐producing RMCE clones, 3.5‐fold for the medium producing plasmid clones, and 3.9‐fold for the BAC clones. Transcript levels of all RMCE clones used in this study were investigated (Mayrhofer et al., 2014) and despite equal levels of transcripts, 2F5‐scFv‐Fc clones had a twofold lower mean specific productivity than 3D6‐scFv‐Fc clones over a period of 10 passages. This is very similar to the herein described difference of 1.7‐fold during a shorter period of time. Mader et al. (2013) described a three to fourfold different specific productivity between 2F5‐ and 3D6‐scFv‐Fc plasmid‐ and BAC‐generated producers, which is in accordance with the results presented in this study. Since both techniques are based on random integration, gene copy numbers and transcript levels can vary between 2F5‐ and 3D6‐scFv‐Fc plasmid‐ and BAC‐derived producers. However, Mader et al. found no correlation between reduced gene copy numbers or levels of transcript and the reduced production rate of 2F5scFv‐Fc compared to the 3D6 antibody fragment. Especially the different expression rates of isogenic RMCE clones lead to the assumption that the established CHO cells are more challenged with the production of the 2F5‐scFv‐Fc antibody fragment compared to the 3D6‐scFv‐Fc. We suppose that physicochemical properties of the nascent polypeptide might cause diminished translational activity or post‐translational processing.

### Thermal Stability

Already previous studies indicated that antibodies which are more stable against thermal unfolding are able to generate clones with improved expression rates (Buchanan et al., 2013; Garber and Demarest, 2007). In our study, DSC measurements revealed that the Fv domains were the least stable and unfolded first at 52.1 ± 0.1 (2F5scFv‐Fc) and 53.5 ± 0.2 (3D6scFv‐Fc). It remains elusive to which extent the thermal stability difference of 1.4°C contributed to the herein observed 1.7 to 3.9‐fold different protein expression but the proportion of our results are in good agreement with the published data.

### Proteomics

In order to understand cellular proteomic differences of the clones producing these antibody fragments, we applied quantitative label‐free LC‐MS proteomic analyses and performed two types of comparisons. According to the population of the included clones, we defined different thresholds for statistical analyses but for both analyses we considered only proteins identified by two or more peptides with an adjusted *P*‐value ≤0.05. For the “2F5 versus 3D6 transgene comparison,” a fold‐change cut‐off of 1.2 was applied since we did not expect severe differences in this comparison of two phenotypically heterogeneous groups. For the “2F5 and 3D6 transgene delivery comparison,” we increased the cut‐off to 1.5 since we compared relatively small and homogenous groups with comparably diverse phenotypes.

#### Transgene Comparison

The overall proteomic comparison of the two groups of 2F5‐scFv‐Fc (*n* = 6) and 3D6‐scFv‐Fc (*n* = 6) producers in triplicates revealed 60 statistically significant differentially expressed proteins. According to the heterogeneity of the population, the recombinant product itself was not identified to be differentially expressed. This is in accordance with previously published intracellular flow cytometry data (Mader et al., 2013; Mayrhofer et al., 2014) showing comparably no severe differences in intracellular product levels between 2F5‐ and 3D6‐sc‐FvFc producers. Therefore the significantly lower specific productivities for 2F5‐scFv‐Fc producers strengthen the assumption that translation and/or ER throughput is decelerated in 2F5‐scFv‐Fc clones. Gene enrichment analysis of the 60 differentially expressed proteins highlighted among others the processes of proliferation and protein folding to be significantly enriched. We found very interesting candidates taking part in several, probably phenotype related, processes. A detailed description of their cellular functions can be found in the supplemental material. The higher abundance of proteins involved in proliferation, apoptosis, and cellular stress (f.i. Lgals3, Hspd1, Hspe1, Mcm5) in the group of the apparently difficult to produce 2F5‐scFv‐Fc clones suggests an adjustment to permanent stressful cellular conditions. Furthermore, the higher abundance of proteins interacting with chromatin and histones in the group of 2F5‐scFv‐Fc producers (f.i. Nat10, Ncl, Ipo5, Mta2) might indicate abnormal regulation of the chromatin structure. The identification of four differentially expressed Glutathione S‐transferases (Gstp2, Gstp1, Gstm6, and Gsto1) higher abundant in the group of 3D6‐scFv‐Fc producers indicates a high impact of S‐glutathionylation in cellular regulation (e.g., stress response and control of cell‐signaling pathways). The higher relative abundances of several proteins involved in folding (Pdia3, Calr, Pdia4, Dnajc7, P4Hb, Hsp90b1, Grpel1) and translation (Eif5a, Eif4a1) in the group of 3D6‐scFv‐Fc producers may be attributed to their overall higher specific production rate. Also the elevated levels of oxidative stress markers (Prdx1, Cat, Txnrd1) may reflect the overall higher ER throughput of 3D6‐scFv‐Fc producers. Reactive oxygen species (ROS) can be produced as by‐products of oxygen‐utilizing enzymatic reactions, such as the mitochondrial respiratory chain. Furthermore, there is accumulating evidence that protein folding, endoplasmic reticulum (ER) stress, and the production of ROS are interlinked (Malhotra and Kaufman, 2007). One very interesting finding was that Sec23Ip, a protein involved in the organization of ER exit sites, was less abundant in 2F5‐scFv‐Fc producers (higher in 3D6‐scFv‐Fc producers). Sec23Ip depletion or overexpression alters ER exit sites morphology and a reduced level delays export from the ER (Ong et al., 2010; Shimoi et al., 2005). Hasegawa et al. (2011) recently reported a striking recombinant CHO phenotype that showed intracellular crystallization of a recombinantly produced model IgG. The study of this particular cell line provided insights in maximum cellular secretory capacity and identified the ER export as a rate‐limiting bottleneck for the particular IgG. These results could be an indication of aggravated ER exit of 2F5‐scFv‐Fc fragments, and Sec23Ip might play a crucial role for the decreased secretory throughput. Hasegawa et al. suggested that an acidic cluster on the surface of the *V*_H_ complementarity determining regions (CDRs) of their model IgG was important for its in vivo crystallization. Taking a closer look at the surface charge of the variable regions of 2F5 and 3D6 (Fig. 2) reveals individual surface charge distributions between the two antibodies. Therefore, we speculate that the surface charge might contribute to the limited secretory throughput.

#### Transgene Delivery Comparison

In the second data evaluation approach, we investigated proteomic changes caused by elevated specific productivities and compared RMCE versus plasmid versus BAC clones for both antibody groups of 2F5‐ and 3D6‐scFv‐Fc producers separately. We were only interested in expression patterns that correlated with the specific productivity or growth rate and therefore neglected identified statistically significant differentially expressed proteins that showed the highest or lowest abundance in the group of plasmid‐derived clones. Several of the identified proteins were already somehow linked to expression in recombinant mammalian cells (f.i. Anxa1, Hsp90ab1, Hspd1, Lgals1, Pdia3, Pdia6, and Vim) (Alete et al., 2005; Baik et al., 2008, 2006; Meleady et al., 2011, 2008; Nissom et al., 2006; Seth et al., 2007; Van Dyk et al., 2003; Wingens et al., 2015; Yee et al., 2008) (Supplemental Tables SII and SIII). We identified 58 proteins plus the immunoglobulin γ‐chain (recombinant product) correlating with specific productivities in both transgene delivery comparisons of 2F5‐ and 3D6‐scFv‐Fc producers. Fourty‐five of these proteins were positively and thirteen negatively correlating to specific productivity. Since these proteins were identified for both transgene delivery comparisons, we consider them to be commonly up‐ or down‐regulated with increasing specific productivities. Gene enrichment analysis revealed cell cycle, vesicle‐mediated transport as well as transport in general to be significantly enriched, which is in accordance with the phenotypical observations. Cell death was also identified to be statistically enriched and may be related to the counterbalance of cellular stress. The highest fold‐change from low producing RMCE clones to high producing BAC clones was identified for the protein leukocyte elastase inhibitor A (Serpinb1a). Serpinb1a negatively regulates the activity of neutrophil proteases (Cooley et al., 2001) and overexpression of Serpinb1 was shown to increase recombinant IgG productivity in CHO (Lin et al., 2015). Cathepsin B, which was also positively correlating with specific productivity, is a protease predominantly present in lysosomes. It is well described that autophagy and lysosomal degradation is activated during ER stress (Cheng and Yang, 2011; Kaminskyy and Zhivotovsky, 2012). These results suggest that independent of the produced scFv‐Fc fragment ER stress increases with increasing specific productivity. Galectin‐1 levels were also increasing in cells with a higher specific productivity in both antibody transgene delivery comparisons. There is evidence that Galectin‐1 is involved in the regulation of cell growth. Higher Galectin‐1 levels in higher producing but slower growing cell lines suggest that Galectin‐1 may be an interesting target for proliferation engineering. We also identified three proteins, gelsolin, Ras‐related protein Rab‐1A, and vesicle‐trafficking protein SEC22b, which are all involved in vesicle mediated transport, to be expressed at increased levels in the higher producing cell lines in both groups. Assuming that secretion engineering might improve the achievable productivities in recombinant CHO cells (Peng and Fussenegger, 2009), these three proteins may be interesting targets for overexpression. Remarkably, we identified fewer proteins that were negatively correlating to specific productivity in both groups. The proteasome‐associated protein ECM29 was identified to be down‐regulated with increasing specific productivity in both groups. It is believed that ECM29 serves as an adaptor for coupling 26 S‐proteasomes to specific cellular compartments (Gorbea et al., 2010). Summarizing the observed results, an increase of lysosomal proteins and a decrease of proteasome associated proteins with increasing specific productivities has been shown. We speculate that at low specific productivities and moderate ER stress levels, the degradation of unfolded proteins is sufficient via the ubiquitin proteasome system. With increasing ER throughput and increasing levels of unfolded proteins the recombinant cells might have to switch to macroautophagy to survive. It has been previously described that the ubiquitin proteasome system and autophagy are interconnected and inhibition of proteasome function leads to activation of autophagy to compensate for the reduced proteasome function (Ding et al., 2007; Korolchuk et al., 2010).

The fact that we identified numerous proteins only in one transgene delivery comparison is another hint that the type of recombinant product itself has an impact on the proteome of recombinant CHO cells.

We identified only two proteins that displayed an opposing trend in the expression pattern comparing the two groups. Both proteins, however, were only slightly changed in one group and only passed the threshold of >1.50‐fold change in the other group. The proteins were Cullin‐associated NEDD8‐dissociated protein 1 (Cand1) as well as protein SON. Cand1 is involved in the regulation of SCF ubiquitin ligases (Chua et al., 2011; Olma and Dikic, 2013). Ubiquitination plays a crucial role in the nuclear factor‐κB (NFκB) pathway, endocytic trafficking, DNA repair, and protein degradation (Grabbe et al., 2011). SON acts as an mRNA splicing cofactor and promotes splicing of many cell‐cycle and DNA‐repair transcripts (Ahn et al., 2011). Since these proteins were the only ones identified that showed an opposing expression pattern comparing the identified proteins positively or negatively correlating to the specific productivity in the transgene delivery comparison of 2F5‐ and 3D6‐scFv‐Fc producers, we believe that these proteins might play a crucial role in cellular regulations leading to the observed phenotypes.

## Conclusion

We could clearly show that distinct recombinant proteins evoke different proteomic responses within the cells. Furthermore, we found indications that the presumably hard to produce 2F5‐scFv‐Fc fragment induces permanent stress within the recombinant CHO cells. The higher abundance of histone and chromatin interacting proteins in the group of 2F5‐scFv‐Fc producers might be a result of abnormal regulation of the chromatin structure. Furthermore, in context with a study published by Hasegawa et al. (2011), the lower levels of Sec23lp may be connected to the decelerated ER‐throughput, and probably caused by the surface charge distribution on the CDRs of the 2F5‐scFv‐Fc .

In the second part of this study, we found evidence that ER stress is increasing with higher specific productivities for both groups of 2F5‐ and 3D6‐scFv‐Fc producers. The presented data also suggest that macroautophagy might play a crucial role for survival of ER‐stressed high‐producers. Interestingly, we could identify two proteins, Cand1 and SON, which showed the opposite correlation to the specific production rate in the two groups. Therefore, these proteins might be some key regulators of specific production rates.

In summary, we could show that the level of specific product secretion largely influences the cell's proteome. More striking is the fact that we could show that the recombinantly produced protein itself considerably influences the cell's physiology. In a consequence, these findings also suggest that host engineering strategies might only work for a subset of recombinant proteins.

This work has been financially supported by Polymun Scientific GmbH, Klosterneuburg, Austria and the University of Natural Resources and Life Sciences, Vienna. WS and PM were also supported by the PhD program “BioToP‐Biomolecular Technology of Proteins” (Austrian Science Fund, FWF Project W1224). We thank Alexander Mader, Andreas Gili, Bernhard Kratzer, Bernhard Prewein, and Martina Hofbauer for their help with cell line development.

## Supporting information

Additional supporting information may be found in the online version of this article at the publisher's web‐site.

**Figure S1**. Clustal sequence alignment of 2F5‐ and 3D6‐scFv‐Fc.
**Figure S2**. Progenesis output: PCA of all differential peptides (*P* ≤ 0.05) used for protein identification.
**Figure S3**. Progenesis output: Normalized abundance of human Ig gamma‐1 chain C region in 2F5‐scFv‐Fc samples (A) and 3D6‐scFv‐Fc samples (B).
**Table S1**. Transgene comparison: 2F5‐scFv‐Fc (*n* = 18) versus 3D6‐scFv‐Fc (*n* = 18) identified differential proteins (*n* = 60); number of peptides used for quantitation ≥2; Anova *P*‐value ≤0.05; fold change ≥1.2×.
**Table S2**. Transgene delivery comparison: 2F5‐scFv‐Fc RMCE (*n* = 6) versus plasmid (*n* = 6) versus BAC (*n* = 6) samples.
**Table S3**. Transgene delivery comparison: 3D6‐scFv‐Fc RMCE (*n* = 6) versus plasmid (*n* = 6) versus BAC (*n* = 6) samples.
**Table S4**. Transgene delivery comparison RMCE (*n* = 6) versus plasmid (*n* = 6) versus BAC (*n* = 6); same expression pattern for 2F5‐scFv‐Fc and 3D6‐scFv‐Fc; combined identified differential proteins (*n* = 58 + 1); number of peptides used for quantitation ≥2; Anova *P*‐value ≤0.05; fold change ≥1.5× in both in‐group comparisons.
**Table S5**. Transgene delivery comparison RMCE (*n* = 6) versus plasmid (*n* = 6) versus BAC (*n* = 6); 2F5‐scFv‐Fc identified differential proteins (*n* = 9) correlating to qP or µ that were not identified in the in‐group comparison of 3D6‐scFv‐Fc at all; number of peptides used for quantitation ≥2; Anova *P*‐value ≤0.05; fold change ≥1.5× in both in‐group comparisons.
**Table S6**. Transgene delivery comparison RMCE (*n* = 6) versus plasmid (*n* = 6) versus BAC (*n* = 6); 3D6 scFv‐Fc identified differential proteins (*n* = 32) correlating to qP or µ that were not identified in the in‐group comparison of 2F5‐scFv‐Fc at all; number of peptides used for quantitation ≥2; Anova *P*‐value ≤0.05; fold change ≥1.5× in both in‐group comparisons.
**Table S7**. Transgene delivery comparison RMCE (*n* = 6) versus plasmid (*n* = 6) versus BAC (*n* = 6); differential expressed proteins correlating with qP or µ showing the opposite expression pattern for 3D6‐scFv‐Fc and 2F5‐scFv‐Fc producers (*n* = 2) (number of peptides used for quantitation ≥2; Anova *P*‐value ≤0.05; fold change ≥1.5× in at least one of the two in‐group comparisons).
**Table S8**. GeneCodis3 output: GOSlim process singular enrichment analysis of gene list (*n* = 60) of proteins identified to be differential between 2F5‐ and 3D6‐scFv‐Fc clones (transgene comparison).
**Table S9**. GeneCodis3 output: GOSlim process singular enrichment of gene list (*n* = 58) of proteins that showed the same expression profile in both transgene delivery comparisons.
