# Supplementary material for: Proteomic differences in recombinant CHO cells producing two similar antibody fragments
Source: Biotechnol Bioeng. 2016 Mar 16;113(9):1902–12. doi: 10.1002/bit.25957 (PMC4985663; doi:10.1002/bit.25957)
Supplement: Supplementary file 1 — Figure S1. Clustal sequence alignment of 2F5‐ and 3D6‐scFv‐Fc. Figure S2. Progenesis output: PCA of all differential peptides (P ≤ 0.05) used for protein identification. Figure S3. Progenesis output: Normalized abundance of human Ig gamma‐1 chain C region in 2F5‐scFv‐Fc samples (A) and 3D6‐scFv‐Fc samples (B). Table S1. Transgene comparison: 2F5‐scFv‐Fc (n = 18) versus 3D6‐scFv‐Fc (n = 18) identified differential proteins (n = 60); number of peptides used for quantitation ≥2; Anova P‐value ≤0.05; fold change ≥1.2×. Table S2. Transgene delivery comparison: 2F5‐scFv‐Fc RMCE (n = 6) versus plasmid (n = 6) versus BAC (n = 6) samples. Table S3. Transgene delivery comparison: 3D6‐scFv‐Fc RMCE (n = 6) versus plasmid (n = 6) versus BAC (n = 6) samples. Table S4. Transgene delivery comparison RMCE (n = 6) versus plasmid (n = 6) versus BAC (n = 6); same expression pattern for 2F5‐scFv‐Fc and 3D6‐scFv‐Fc; combined identified differential proteins (n = 58 + 1); number of peptides used for quantitation ≥2; Anova P‐value ≤0.05; fold change ≥1.5× in both in‐group comparisons. Table S5. Transgene delivery comparison RMCE (n = 6) versus plasmid (n = 6) versus BAC (n = 6); 2F5‐scFv‐Fc identified differential proteins (n = 9) correlating to qP or µ that were not identified in the in‐group comparison of 3D6‐scFv‐Fc at all; number of peptides used for quantitation ≥2; Anova P‐value ≤0.05; fold change ≥1.5× in both in‐group comparisons. Table S6. Transgene delivery comparison RMCE (n = 6) versus plasmid (n = 6) versus BAC (n = 6); 3D6 scFv‐Fc identified differential proteins (n = 32) correlating to qP or µ that were not identified in the in‐group comparison of 2F5‐scFv‐Fc at all; number of peptides used for quantitation ≥2; Anova P‐value ≤0.05; fold change ≥1.5× in both in‐group comparisons. Table S7. Transgene delivery comparison RMCE (n = 6) versus plasmid (n = 6) versus BAC (n = 6); differential expressed proteins correlating with qP or µ showing the opposite expression pattern for 3D [file BIT-113-1902-s001.docx]

# **Proteomic differences in recombinant CHO cells producing two similar antibody fragments**

# **Supplemental material**

**scFv-Fc fragments**

2F5scFv-Fc-aa RITLKESGPPLVKPTQTLTLTCSFSGFSLSDFGVGVGWIRQPPGKALEWLAII-YSDDDK

3D6scFv-Fc-aa EVQLVESGGGLVQPGRSLRLSCAASGFTFNDYA--MHWVRQAPGKGLEWVSGISWDSSSI

.: * *** **:* ::* *:*: ***::.*:. : *:** ***.***:: * :....

2F5scFv-Fc-aa RYSPSLNTRLTITKDTSKNQVVLVMTRVSPVDTATYFCAHRRGPTTLFGVPIARGPVNAM

3D6scFv-Fc-aa GYADSVKGRFTISRDNAKNSLYLQMNSLRAEDMALYYCVKGRDYYDSGG-----YFTVAF

*: *:: *:**::*.:**.: * *. : * * *:*.: * * . *:

2F5scFv-Fc-aa DVWGQGITVTISSGGGGSGGGGSGGGGSALQLTQSPSSLSASVGDRITITCRASQGVTSA

3D6scFv-Fc-aa DIWGQGTMVTVSSGGGGSGGGGSGGGGSDIQMTQSPSTLSASVGDRVTITCRASQSISRW

*:**** **:***************** :*:*****:********:********.::

2F5scFv-Fc-aa LAWYRQKPGSPPQLLIYDASSLESGVPSRFSGSGSGTEFTLTISTLRPEDFATYYCQQLH

3D6scFv-Fc-aa LAWYQQKPGKVPKLLIYKASSLESGVPSRFSGSGSGTEFTLTISSLQPDDFATYYCQQYN

****:****. *:****.**************************:*:*:********* .

2F5scFv-Fc-aa FYPHTFGGGTRVDVREPKSSDKTHTCPPCPAPELLGGPSVFLFPPKPKDTLMISRTPEVT

3D6scFv-Fc-aa SY--SFGPGTKVDIKEPKSSDKTHTCPPCPAPELLGGPSVFLFPPKPKDTLMISRTPEVT

* :** **:**::*********************************************

2F5scFv-Fc-aa CVVVDVSHEDPEVKFNWYVDGVEVHNAKTKPREEQYNSTYRVVSVLTVLHQDWLNGKEYK

3D6scFv-Fc-aa CVVVDVSHEDPEVKFNWYVDGVEVHNAKTKPREEQYNSTYRVVSVLTVLHQDWLNGKEYK

************************************************************

2F5scFv-Fc-aa CKVSNKALPAPIEKTISKAKGQPREPQVYTLPPSRDELTKNQVSLTCLVKGFYPSDIAVE

3D6scFv-Fc-aa CKVSNKALPAPIEKTISKAKGQPREPQVYTLPPSRDELTKNQVSLTCLVKGFYPSDIAVE

************************************************************

2F5scFv-Fc-aa WESNGQPENNYKTTPPVLDSDGSFFLYSKLTVDKSRWQQGNVFSCSVMHEALHNHYTQKS

3D6scFv-Fc-aa WESNGQPENNYKTTPPVLDSDGSFFLYSKLTVDKSRWQQGNVFSCSVMHEALHNHYTQKS

************************************************************

2F5scFv-Fc-aa LSLSPGK

3D6scFv-Fc-aa LSLSPGK

*******

**Supplemental Figure 1:** Clustal sequence alignment of 2F5- and 3D6-scFv-Fc.

**Principal component analysis (PCA)**


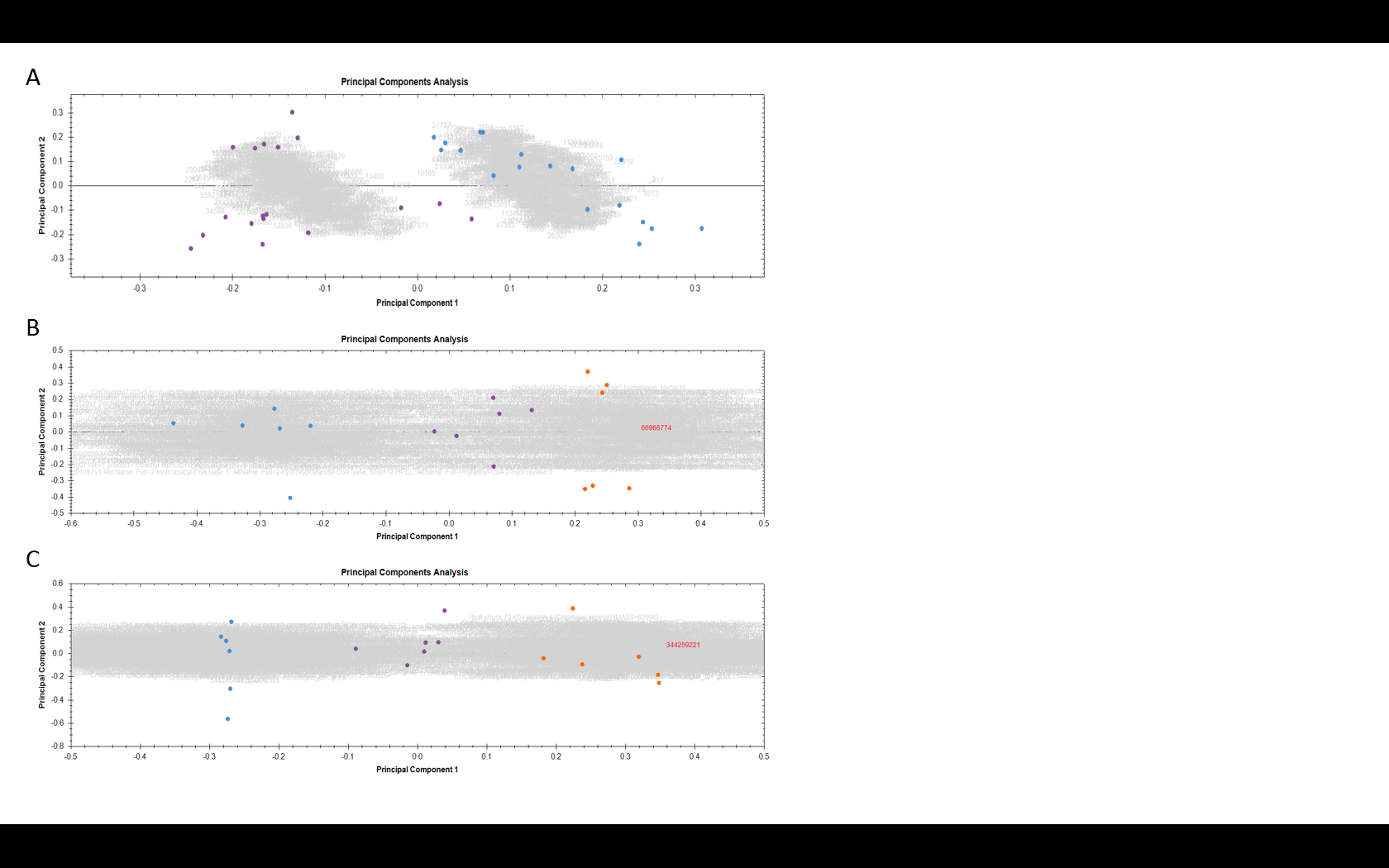


**Supplemental Figure 2:** Progenesis output: PCA of all differential peptides (p≤0.05) used for protein identification; A: transgene comparison of 3D6 (n=18, blue) versus 2F5 (n=18, purple) samples; B: 2F5 transgene delivery comparison of RMCE (n=6, blue) versus Plasmid (n=6 purple) versus BAC (n=6, orange) samples; C: 3D6 transgene delivery comparison of RMCE (n=6, blue) versus Plasmid (n=6 purple) versus BAC (n=6, orange) samples.

**Recombinant product identified in proteomic analysis**


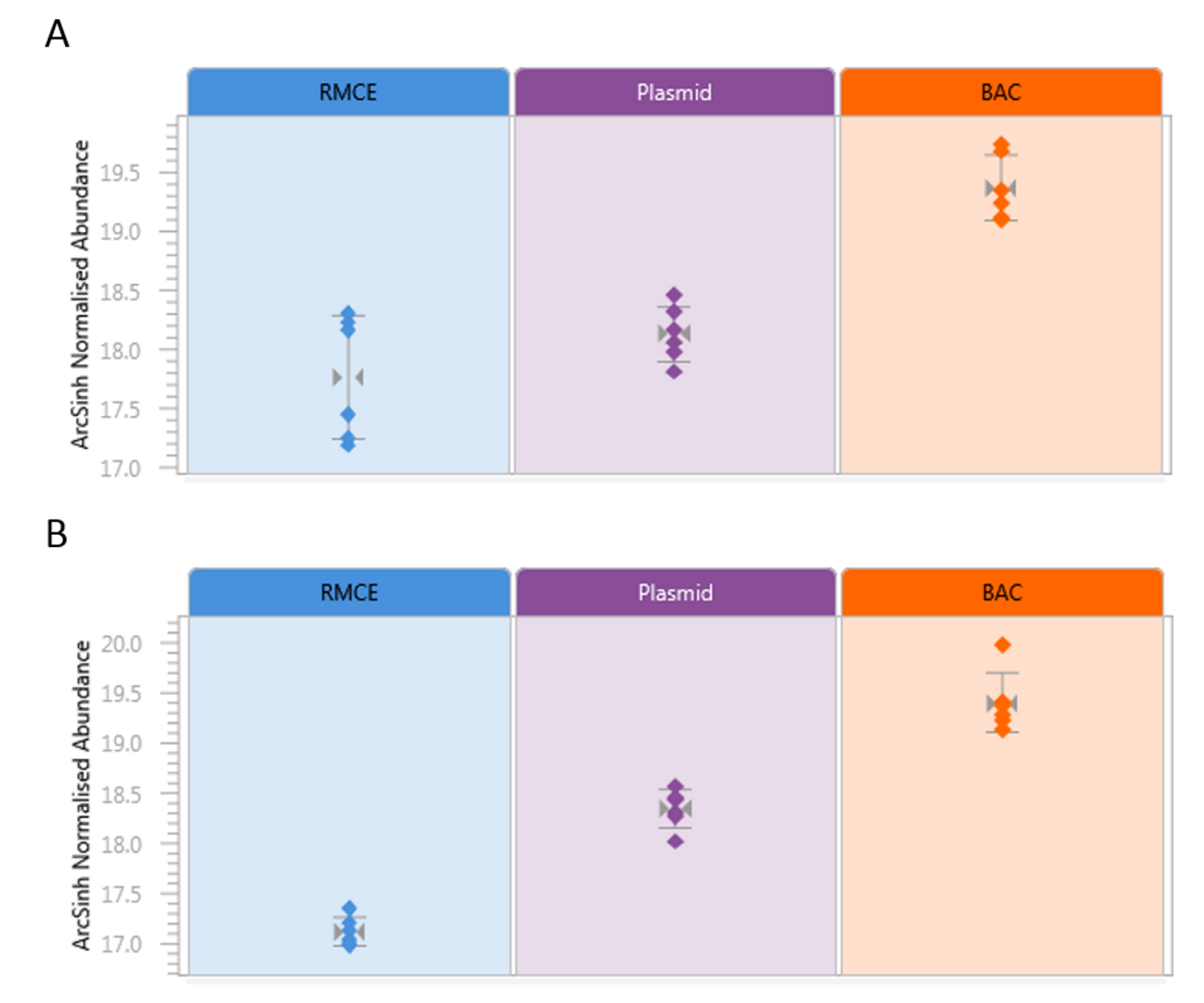


**Supplemental Figure 3:** Progenesis output: Normalised abundance of human Ig gamma-1 chain C region in 2F5-scFv-Fc samples (A) and 3D6-scFv-Fc samples (B).

**Identified DE proteins (transgene comparison)**

**Supplemental Table 1:** Transgene comparison: 2F5-scFv-Fc (n=18) versus 3D6-scFv-Fc (n=18) identified differential proteins (n=60); number of peptides used for quantitation ≥ 2; Anova p Value ≤ 0.05; fold change **≥ 1.2x**. PCA of features used for identifications are shown in supplemental figure 2, A. Table is sorted by fold-change.

| **Description** | **Gene ID** | **Peptides used for quant.** | **Anova (p)** | **Fold change** | **Highest mean** |
| --- | --- | --- | --- | --- | --- |
| Galectin-3 | Lgals3 | 2 | 0.00 | 1.75 | 2F5 |
| Calcium/calmodulin-dependent protein kinase type II subunit delta | Camk2d | 2 | 0.00 | 1.59 | 2F5 |
| DNA replication licensing factor MCM5 | Mcm5 | 2 | 0.01 | 1.58 | 2F5 |
| 60 kDa heat shock protein, mitochondrial | Hspd1 | 10 | 0.00 | 1.50 | 2F5 |
| N-acetyltransferase 10 | Nat10 | 2 | 0.02 | 1.49 | 2F5 |
| Nucleolin | Ncl | 7 | 0.00 | 1.43 | 2F5 |
| 10 kDa heat shock protein, mitochondrial | Hspe1 | 5 | 0.00 | 1.43 | 2F5 |
| Heme oxygenase 2 | Hmox2 | 3 | 0.02 | 1.42 | 2F5 |
| Splicing factor 3B subunit 1 | Sf3b1 | 3 | 0.00 | 1.36 | 2F5 |
| NADPH:adrenodoxin oxidoreductase, mitochondrial | Fdxr | 3 | 0.01 | 1.35 | 2F5 |
| Tropomyosin alpha-4 chain | Tpm4 | 2 | 0.02 | 1.35 | 2F5 |
| Intracellular adhesion molecule 1 | Icam1 | 2 | 0.04 | 1.35 | 2F5 |
| Putative ribosomal RNA methyltransferase NOP2 | Nop2 | 2 | 0.01 | 1.29 | 2F5 |
| Lanosterol synthase | Lss | 3 | 0.00 | 1.28 | 2F5 |
| BRI3-binding protein | Bri3bp | 2 | 0.00 | 1.28 | 2F5 |
| Importin-5 | Ipo5 | 2 | 0.02 | 1.27 | 2F5 |
| Drebrin-like | Dbnl | 3 | 0.03 | 1.26 | 2F5 |
| Antigen KI-67 | Mki67 | 2 | 0.02 | 1.25 | 2F5 |
| Nuclear mitotic apparatus protein 1 | Numa1 | 2 | 0.00 | 1.23 | 2F5 |
| Filamin-B | Flnb | 2 | 0.04 | 1.23 | 2F5 |
| Metastasis-associated protein MTA2 | Mta2 | 2 | 0.01 | 1.22 | 2F5 |
| Annexin A2 | Anxa2 | 2 | 0.02 | 1.22 | 2F5 |
| Cysteine desulfurase, mitochondrial | Nfs1 | 2 | 0.00 | 1.22 | 2F5 |
| Serine/arginine repetitive matrix protein 2 | Srrm2 | 3 | 0.00 | 1.22 | 2F5 |
| Aldehyde oxidase | Aox1 | 2 | 0.01 | 1.21 | 2F5 |
| Succinyl-CoA:3-ketoacid-coenzyme A transferase 1, mitochondrial | Oxct1 | 3 | 0.00 | 1.21 | 2F5 |
| DNA topoisomerase 1 | Top1 | 2 | 0.01 | 1.21 | 2F5 |
| Glutathione S-transferase P 2 | Gstp2 | 3 | 0.00 | 2.74 | 3D6 |
| Glutathione S-transferase P 1 | Gstp1 | 3 | 0.00 | 2.19 | 3D6 |
| Peroxiredoxin-1 | Prdx1 | 6 | 0.00 | 1.76 | 3D6 |
| Sulfide:quinone oxidoreductase, mitochondrial | Sqrdl | 2 | 0.03 | 1.75 | 3D6 |
| Golgi-associated plant pathogenesis-related protein 1 | Glipr2 | 2 | 0.00 | 1.64 | 3D6 |
| ^9^Protein disulfide-isomerase A3 | Pdia3 | 2 | 0.01 | 1.58 | 3D6 |
| Catalase | Cat | 2 | 0.00 | 1.52 | 3D6 |
| Calreticulin | Calr | 4 | 0.00 | 1.47 | 3D6 |
| Basement membrane-specific heparan sulfate proteoglycan core protein | Hspg2 | 3 | 0.01 | 1.45 | 3D6 |
| Von Willebrand factor A domain-containing protein 5A | Vwa5a | 2 | 0.03 | 1.42 | 3D6 |
| Hydroxymethylglutaryl-CoA lyase, mitochondrial | Hmgcl | 2 | 0.00 | 1.39 | 3D6 |
| SEC23-interacting protein | Sec23Ip | 2 | 0.00 | 1.38 | 3D6 |
| ATP-citrate synthase | Acly | 2 | 0.00 | 1.38 | 3D6 |
| Fumarylacetoacetase | Fah | 3 | 0.01 | 1.36 | 3D6 |
| Glutamate--cysteine ligase regulatory subunit | Gclm | 4 | 0.02 | 1.35 | 3D6 |
| Alpha-enolase | Eno1 | 11 | 0.00 | 1.33 | 3D6 |
| Protein disulfide-isomerase A4 | Pdia4 | 3 | 0.00 | 1.33 | 3D6 |
| Pyruvate carboxylase, mitochondrial | Pcx | 4 | 0.01 | 1.31 | 3D6 |
| Thioredoxin reductase 1, cytoplasmic | Txnrd1 | 3 | 0.02 | 1.29 | 3D6 |
| Glucose-6-phosphate 1-dehydrogenase | G6Pdx | 4 | 0.00 | 1.28 | 3D6 |
| Glutathione S-transferase Mu 6 | Gstm6 | 4 | 0.01 | 1.28 | 3D6 |
| Eukaryotic translation initiation factor 5A-1 | Eif5a | 2 | 0.01 | 1.28 | 3D6 |
| Nucleobindin-2 | Nucb2 | 2 | 0.00 | 1.27 | 3D6 |
| Eukaryotic initiation factor 4A-I | Eif4a1 | 3 | 0.00 | 1.26 | 3D6 |
| Glutathione S-transferase omega-1 isoform 1 | Gsto1 | 3 | 0.00 | 1.26 | 3D6 |
| Alcohol dehydrogenase (NADP+) | Adh5 | 2 | 0.00 | 1.26 | 3D6 |
| DnaJ homolog subfamily C member 7 | Dnajc7 | 2 | 0.02 | 1.23 | 3D6 |
| Importin subunit beta-1 | Kpnb1 | 6 | 0.00 | 1.23 | 3D6 |
| Protein disulfide-isomerase | P4Hb | 3 | 0.02 | 1.23 | 3D6 |
| Endoplasmin | Hsp90b1 | 5 | 0.01 | 1.23 | 3D6 |
| GrpE protein homolog 1, mitochondrial | Grpel1 | 3 | 0.00 | 1.21 | 3D6 |
| Nucleoside diphosphate kinase A | Nme1 | 2 | 0.02 | 1.21 | 3D6 |
| Chloride intracellular channel protein 4 | Clic4 | 3 | 0.01 | 1.21 | 3D6 |

**Discussion of identified DE proteins (transgene comparison)**

Galectin-3 (Lgals3), which was found to be present in higher amounts in 2F5-scFv-Fc producers, is an interesting protein that is involved in several cellular processes like adhesion, cell cycle progression and apoptosis as well as inflammatory processes (Dumic et al. 2006). In the cytoplasm Galectin-3 interacts with the apoptosis repressor Bcl-2; in the nucleus, Galectin-3 is a required pre-mRNA splicing factor (Haudek et al. 2010). Two other proteins with elevated levels in the group of 2F5-scFv-Fc producers are heat shock protein 60 (Hsp60/Hspd1) and its co-chaperone heat shock protein 10 (Hsp10/Hspe1). Amongst other heat shock proteins, Hspd1 was shown to be important for cellular survival under stressful conditions (Rossi et al. 2002). Another versatile higher abundant protein in the group of 2F5-scFv-Fc producers is DNA replication licensing factor MCM5 (Mcm5). Mcm5 is one of the key proteins in DNA replication initiation but it was also shown to be essential in Stat1-mediated transcriptional activation (Snyder et al. 2005). N-acetyltransferase 10 (Nat10) was as well found to be higher in 2F5-scFv-Fc producers. Nat10 influences histone acetylation and up-regulates telomerase activity through transactivation of hTERT promoter (Lv et al. 2003). Furthermore, it was described that Nat10 is involved in DNA damage response and increases resistance to genotoxicity (Liu et al. 2007). The protein nucleolin was found to be 1.43-fold higher in the 2F5 producers. As a DNA-binding protein, nucleolin triggers chromatin decondensation, facilitates transcription and modulates DNA replication (Angelov et al. 2006). Nucleolin is predominantly present in the nucleolus, where it also interacts with rRNA and assists in its maturation and processing (Ginisty et al. 1998). In the cytoplasm, nucleolin interacts with mature mRNAs. Recently, nucleolin was shown to associate with several mRNAs encoding proteins with roles in cell growth and proliferation, including Bcl-2, p53, cyclin I, and Akt1 (Abdelmohsen et al. 2011). Importin-5 which was also found to be slightly higher in 2F5-scFv-Fc producers mediates the import of ribosomal proteins and core histones into the nucleus (Baake et al. 2001; Jäkel and Görlich 1998). Another very interesting protein up regulated in 2F5-scFv-Fc producers is Mta2, which is part of NuRD, the nucleosome remodelling and deacetylase complex (Xue et al. 1998). NuRD prevents the accumulation of spontaneous DNA damage and regulates apoptotic responses through p53 and p21 (Smeenk et al. 2010). Interestingly we could identify four Glutathione S-transferases (Gstp2, Gstp1, Gstm6 and Gsto1) to be less abundant in 2F5-scFv-Fc producers (higher in 3D6-scFv-Fc producers). S-glutathionylation has an impact on several proteins including signalling proteins, transcription factors as well as heat shock proteins (Tew et al. 2011). Peroxiredoxin-1 (Prdx1) levels were also higher in 3D6-scFv-Fc producing CHO cells. Peroxiredoxin-1 has an important role in maintaining the redox state *in vivo*. Furthermore, Prdx1 and other members of the Prdx family, are also involved in regulating growth factor signalling pathways (Rhee et al. 2003). Golgi-associated plant pathogenesis-related protein 1 (Glioma pathogenesis-related protein 2 (Glipr2)) was also higher in abundance in 3D6-scFv-Fc samples. Overexpression of Glipr2 in human kidney 2 (HK-2) cells suggested that it has an ERK 1/2 activating function. ERK 1/2 signalling itself plays a central role in cell proliferation and apoptosis (Mebratu and Tesfaigzi 2009). Catalase, which showed elevated expression in 3D6 samples, is an antioxidant enzyme found in nearly all living organisms that are exposed to oxygen and catalyzes the reduction of H_2_O_2_ to water (Kirkman and Gaetani 2007) and is also a marker of oxidative stress. Thioredoxin reductase 1, which was also slightly increased in 3D6 producers, is another indicator of oxidative stress. Reactive oxygen species (ROS) can be produced as by-products of oxygen-utilizing enzymatic reactions, such as the mitochondrial respiratory chain. Furthermore, there is accumulating evidence that protein folding, endoplasmic reticulum (ER) stress and the production of ROS are interlinked (Malhotra and Kaufman 2007). One very interesting finding was that Sec23Ip, a protein involved in the organization of ER exit sites, was less abundant in 2F5-scFv-Fc producers (higher in 3D6-scFv-Fc producers). Sec23Ip depletion or overexpression alters ER exit sites morphology and a reduced level delays export from the ER (Ong et al. 2010; Shimoi et al. 2005). Furthermore, we detected several proteins involved in folding (Pdia3, Calr, Pdia4, Dnajc7, P4Hb, Hsp90b1, Grpel1) and translation (Eif5a, Eif4a1) to be higher abundant in 3D6-scFv-Fc producers.

**Identified DE proteins (transgene delivery comparison)**

**Supplemental Table 2:** Transgene delivery comparison: 2F5-scFv-Fc RMCE (n=6) versus Plasmid (n=6) versus BAC (n=6) samples. Identified differentially expressed proteins (n=109) between RMCE versus Plasmid versus BAC correlating to qP and µ (highest BAC and lowest RMCE or vice versa); number of peptides used for quantitation ≥ 2; Anova p Value ≤ 0.05; fold change **≥ 1.5x**. PCA of features used for identification are shown in supplemental figure 2, B. Table is sorted by fold-change and positive or negative correlation with qP.

| **Description** | **Peptides used for quant.** | **Anova (p)** | **Fold change** | **Highest mean** | **Lowest mean** |
| --- | --- | --- | --- | --- | --- |
| Leukocyte elastase inhibitor A | 7 | 0,0000 | 5,77 | BAC | RMCE |
| Cornifin-A | 3 | 0,0115 | 5,41 | BAC | RMCE |
| ^2,4,6^Galectin-1 | 9 | 0,0000 | 4,91 | BAC | RMCE |
| NADH-cytochrome b5 reductase 1 | 2 | 0,0002 | 3,76 | BAC | RMCE |
| Cathepsin B | 7 | 0,0001 | 3,51 | BAC | RMCE |
| Hypothetical protein LOC100752925 | 2 | 0,0000 | 3,42 | BAC | RMCE |
| Myosin-9 | 4 | 0,0021 | 3,22 | BAC | RMCE |
| Alpha-N-acetylgalactosaminidase | 4 | 0,0000 | 3,20 | BAC | RMCE |
| Hypothetical protein LOC100752970 | 3 | 0,0000 | 3,12 | BAC | RMCE |
| Cysteine-rich with EGF-like domain protein 2 | 3 | 0,0000 | 3,01 | BAC | RMCE |
| Plectin | 2 | 0,0004 | 2,77 | BAC | RMCE |
| Actin-related protein 2/3 complex subunit 1B | 2 | 0,0061 | 2,71 | BAC | RMCE |
| ^9^Actin-related protein 2/3 complex subunit 2 | 6 | 0,0004 | 2,67 | BAC | RMCE |
| ^10^Gelsolin | 3 | 0,0000 | 2,57 | BAC | RMCE |
| Heat shock protein beta-1 | 2 | 0,0117 | 2,51 | BAC | RMCE |
| Isoleucyl-tRNA synthetase, mitochondrial | 5 | 0,0000 | 2,41 | BAC | RMCE |
| ^2,4,6,9^Vimentin | 24 | 0,0017 | 2,31 | BAC | RMCE |
| Myoferlin | 2 | 0,0003 | 2,28 | BAC | RMCE |
| ^4^Macrophage-capping protein | 5 | 0,0000 | 2,27 | BAC | RMCE |
| Glutathione peroxidase 1 | 2 | 0,0140 | 2,22 | BAC | RMCE |
| Zyxin | 3 | 0,0004 | 2,17 | BAC | RMCE |
| Glycerol-3-phosphate dehydrogenase 1-like protein | 2 | 0,0021 | 2,17 | BAC | RMCE |
| Transgelin-2 | 4 | 0,0004 | 2,15 | BAC | RMCE |
| Succinyl-CoA ligase [ADP-forming] subunit beta, mitochondrial | 5 | 0,0000 | 2,13 | BAC | RMCE |
| Pyridoxal-dependent decarboxylase domain-containing protein 1 | 2 | 0,0001 | 2,11 | BAC | RMCE |
| LIM domain and actin-binding protein 1 | 4 | 0,0028 | 2,10 | BAC | RMCE |
| Coronin-1B | 8 | 0,0000 | 2,08 | BAC | RMCE |
| Myosin-10 | 3 | 0,0010 | 2,05 | BAC | RMCE |
| Rho GTPase-activating protein 1 | 4 | 0,0000 | 2,05 | BAC | RMCE |
| Ras-related protein Rab-1A | 2 | 0,0000 | 2,01 | BAC | RMCE |
| Translocation protein SEC62 | 2 | 0,0001 | 2,01 | BAC | RMCE |
| HIV Tat-specific factor 1 homolog | 2 | 0,0119 | 1,97 | BAC | RMCE |
| Actin-related protein 2 | 4 | 0,0008 | 1,97 | BAC | RMCE |
| ^9^ATP-citrate synthase | 4 | 0,0001 | 1,97 | BAC | RMCE |
| Transmembrane protein 43 | 2 | 0,0000 | 1,96 | BAC | RMCE |
| Pyruvate carboxylase, mitochondrial | 2 | 0,0144 | 1,95 | BAC | RMCE |
| ^6^Peptidyl-prolyl cis-trans isomerase B | 7 | 0,0001 | 1,94 | BAC | RMCE |
| Filamin-B | 29 | 0,0000 | 1,91 | BAC | RMCE |
| Ras-related protein Ral-B | 2 | 0,0025 | 1,91 | BAC | RMCE |
| ^9^Microtubule-associated protein 1B | 2 | 0,0000 | 1,89 | BAC | RMCE |
| Serine/threonine-protein phosphatase 2A 55 kDa regulatory subunit B alpha isoform | 3 | 0,0002 | 1,89 | BAC | RMCE |
| Nucleobindin-2 | 3 | 0,0001 | 1,85 | BAC | RMCE |
| Estradiol 17-beta-dehydrogenase 12 | 3 | 0,0000 | 1,85 | BAC | RMCE |
| Sulfated glycoprotein 1 | 10 | 0,0005 | 1,84 | BAC | RMCE |
| 4-trimethylaminobutyraldehyde dehydrogenase | 2 | 0,0000 | 1,84 | BAC | RMCE |
| Actin-related protein 3 | 5 | 0,0030 | 1,83 | BAC | RMCE |
| Acid ceramidase | 2 | 0,0002 | 1,77 | BAC | RMCE |
| Src substrate cortactin | 6 | 0,0016 | 1,76 | BAC | RMCE |
| Glutathione S-transferase Mu 7 | 2 | 0,0004 | 1,76 | BAC | RMCE |
| Myosin regulatory light chain 12B | 2 | 0,0067 | 1,75 | BAC | RMCE |
| Destrin | 3 | 0,0008 | 1,75 | BAC | RMCE |
| Reticulocalbin-3 | 3 | 0,0021 | 1,73 | BAC | RMCE |
| Guanine nucleotide-binding protein G(I)/G(S)/G(O) subunit gamma-12 | 2 | 0,0000 | 1,70 | BAC | RMCE |
| NADH-cytochrome b5 reductase 3 | 3 | 0,0000 | 1,69 | BAC | RMCE |
| Vesicle-trafficking protein SEC22b | 3 | 0,0028 | 1,69 | BAC | RMCE |
| ^1^Fumarate hydratase | 3 | 0,0000 | 1,67 | BAC | RMCE |
| UPF0556 protein C19orf10-like | 2 | 0,0012 | 1,67 | BAC | RMCE |
| MHC class I antigen Hm1-C4 | 3 | 0,0176 | 1,66 | BAC | RMCE |
| LIM and SH3 domain protein 1 | 8 | 0,0000 | 1,66 | BAC | RMCE |
| Receptor expression-enhancing protein 5 | 2 | 0,0000 | 1,66 | BAC | RMCE |
| Annexin A11 | 5 | 0,0009 | 1,66 | BAC | RMCE |
| Long-chain specific acyl-CoA dehydrogenase, mitochondrial | 8 | 0,0016 | 1,64 | BAC | RMCE |
| Calmodulin | 3 | 0,0008 | 1,62 | BAC | RMCE |
| 3-keto-steroid reductase | 2 | 0,0002 | 1,61 | BAC | RMCE |
| V-type proton ATPase subunit G 1 | 2 | 0,0005 | 1,58 | BAC | RMCE |
| V-type proton ATPase subunit B, brain isoform | 2 | 0,0005 | 1,58 | BAC | RMCE |
| Septin-11 | 2 | 0,0011 | 1,57 | BAC | RMCE |
| Dolichyl-diphosphooligosaccharide--protein glycosyltransferase subunit STT3A | 3 | 0,0000 | 1,57 | BAC | RMCE |
| Vesicle-associated membrane protein-associated protein A | 5 | 0,0108 | 1,56 | BAC | RMCE |
| Protein canopy-like 2 | 3 | 0,0000 | 1,56 | BAC | RMCE |
| AP-2 complex subunit mu-like isoform 2 [Monodelphis domestica] | 2 | 0,0001 | 1,53 | BAC | RMCE |
| Golgi apparatus protein 1 | 4 | 0,0018 | 1,53 | BAC | RMCE |
| Bone marrow stromal antigen 2 | 3 | 0,0441 | 1,52 | BAC | RMCE |
| Prostaglandin E synthase 3 | 3 | 0,0053 | 5,14 | RMCE | BAC |
| Heme oxygenase 1 | 7 | 0,0002 | 3,41 | RMCE | BAC |
| Lipoprotein lipase | 2 | 0,0000 | 2,99 | RMCE | BAC |
| Nucleosome-binding protein 1 | 3 | 0,0060 | 2,80 | RMCE | BAC |
| Glutamate--cysteine ligase regulatory subunit | 5 | 0,0000 | 2,48 | RMCE | BAC |
| ^7^Macrophage migration inhibitory factor | 2 | 0,0084 | 2,41 | RMCE | BAC |
| Exportin-5 | 2 | 0,0000 | 2,33 | RMCE | BAC |
| Phosphoribosylformylglycinamidine synthase | 2 | 0,0000 | 2,32 | RMCE | BAC |
| Anamorsin | 3 | 0,0000 | 2,31 | RMCE | BAC |
| Intracellular adhesion molecule 1 | 8 | 0,0000 | 2,30 | RMCE | BAC |
| carbonyl reductase | 2 | 0,0007 | 2,21 | RMCE | BAC |
| Proteasome-associated protein ECM29-like | 2 | 0,0028 | 2,21 | RMCE | BAC |
| Spliceosome RNA helicase BAT1 | 4 | 0,0025 | 2,14 | RMCE | BAC |
| ^7^Thioredoxin reductase 1, cytoplasmic | 7 | 0,0281 | 1,97 | RMCE | BAC |
| Protein phosphatase 1G | 3 | 0,0004 | 1,91 | RMCE | BAC |
| Aldehyde dehydrogenase, mitochondrial | 6 | 0,0006 | 1,91 | RMCE | BAC |
| Lamina-associated polypeptide 2, isoforms alpha/zeta | 8 | 0,0000 | 1,89 | RMCE | BAC |
| Ribose-phosphate pyrophosphokinase 1 | 3 | 0,0000 | 1,87 | RMCE | BAC |
| Protein SON | 2 | 0,0006 | 1,79 | RMCE | BAC |
| ^9^Heat shock protein 75 kDa, mitochondrial | 7 | 0,0000 | 1,78 | RMCE | BAC |
| Proliferating cell nuclear antigen | 3 | 0,0002 | 1,71 | RMCE | BAC |
| Thioredoxin-like protein 1 | 4 | 0,0001 | 1,69 | RMCE | BAC |
| Basic leucine zipper and W2 domain-containing protein 2 | 5 | 0,0000 | 1,68 | RMCE | BAC |
| Flavin reductase | 10 | 0,0183 | 1,66 | RMCE | BAC |
| Cullin-4B | 2 | 0,0035 | 1,63 | RMCE | BAC |
| ^7^Glutathione S-transferase P 2 | 2 | 0,0014 | 1,63 | RMCE | BAC |
| 6-phosphogluconate dehydrogenase, decarboxylating | 9 | 0,0001 | 1,62 | RMCE | BAC |
| Nuclear autoantigenic sperm protein | 6 | 0,0005 | 1,61 | RMCE | BAC |
| Proliferation-associated protein 2G4 | 10 | 0,0000 | 1,59 | RMCE | BAC |
| UV excision repair protein RAD23 homolog B | 2 | 0,0002 | 1,58 | RMCE | BAC |
| BUB3 budding uninhibited by benzimidazoles 3 homolog (yeast), isoform CRA_c | 4 | 0,0001 | 1,58 | RMCE | BAC |
| Drebrin | 6 | 0,0001 | 1,57 | RMCE | BAC |
| Eukaryotic translation initiation factor 3 subunit J | 3 | 0,0026 | 1,57 | RMCE | BAC |
| ^2,3,9^Heat shock protein HSP 90-beta-like | 34 | 0,0001 | 1,56 | RMCE | BAC |
| GMP synthase [glutamine-hydrolyzing] | 3 | 0,0000 | 1,55 | RMCE | BAC |
| Large proline-rich protein BAT3 | 2 | 0,0002 | 1,53 | RMCE | BAC |

^1-10^ also mentioned in: ^1^Alete et al., 2005; ^2^Baik et al., 2006; ^3^Baik et al., 2008; ^4^Meleady et al., 2008; ^5^Meleady et al., 2011; ^6^Nissom et al., 2006; ^7^Seth et al., 2007; ^8^Van Dyk et al., 2003; ^9^Wingens et al., 2015; ^10^Yee et al., 2008

**Supplemental Table 3:** Transgene delivery comparison: 3D6-scFv-Fc RMCE (n=6) versus Plasmid (n=6) versus BAC (n=6) samples. Identified differentially expressed proteins (n=212) between RMCE versus Plasmid versus BAC correlating to qP and µ (highest BAC and lowest RMCE or vice versa); number of peptides used for quantitation ≥ 2; Anova p value ≤ 0.05; fold change **≥ 1.5x**. PCA of features used for identification are shown in supplemental figure 2, C. Table is sorted by fold-change and positive or negative correlation with qP.

| Description | Peptides used for quant. | Anova (p) | **Fold change** | Highest mean | Lowest mean |
| --- | --- | --- | --- | --- | --- |
| Leukocyte elastase inhibitor A | 8 | 0,0000 | 18,04 | BAC | RMCE |
| Pantetheinase | 3 | 0,0000 | 6,84 | BAC | RMCE |
| Hypothetical protein LOC100752925 | 2 | 0,0000 | 5,54 | BAC | RMCE |
| Cathepsin B | 6 | 0,0000 | 5,14 | BAC | RMCE |
| ^2,4,6^Galectin-1 | 8 | 0,0000 | 4,81 | BAC | RMCE |
| Myoferlin | 2 | 0,0000 | 4,58 | BAC | RMCE |
| Hypothetical protein LOC100752970 | 3 | 0,0000 | 4,25 | BAC | RMCE |
| Sulfide:quinone oxidoreductase, mitochondrial | 2 | 0,0000 | 4,21 | BAC | RMCE |
| ^4^Macrophage-capping protein | 5 | 0,0000 | 4,17 | BAC | RMCE |
| Cysteine-rich with EGF-like domain protein 2 | 3 | 0,0000 | 4,16 | BAC | RMCE |
| ^10^Trifunctional enzyme subunit beta, mitochondrial | 5 | 0,0000 | 4,02 | BAC | RMCE |
| Acyl-CoA synthetase family member 2, mitochondrial | 2 | 0,0000 | 3,76 | BAC | RMCE |
| NADH-cytochrome b5 reductase 1 | 2 | 0,0000 | 3,65 | BAC | RMCE |
| UTP--glucose-1-phosphate uridylyltransferase | 3 | 0,0000 | 3,59 | BAC | RMCE |
| ^10^Trifunctional enzyme subunit alpha, mitochondrial | 11 | 0,0000 | 3,51 | BAC | RMCE |
| Protein-glutamine gamma-glutamyltransferase 2 | 3 | 0,0000 | 3,50 | BAC | RMCE |
| Delta-1-pyrroline-5-carboxylate dehydrogenase, mitochondrial | 2 | 0,0000 | 3,45 | BAC | RMCE |
| Acyl-CoA-binding protein | 2 | 0,0001 | 3,10 | BAC | RMCE |
| Epididymal secretory protein E1 | 2 | 0,0001 | 2,90 | BAC | RMCE |
| LIM domain and actin-binding protein 1 | 5 | 0,0000 | 2,83 | BAC | RMCE |
| ^10^LDLR chaperone MESD | 4 | 0,0007 | 2,78 | BAC | RMCE |
| Cysteine and glycine-rich protein 1 | 5 | 0,0000 | 2,77 | BAC | RMCE |
| Plectin | 3 | 0,0004 | 2,74 | BAC | RMCE |
| 60S acidic ribosomal protein P2 | 2 | 0,0233 | 2,74 | BAC | RMCE |
| Cathepsin Z | 9 | 0,0000 | 2,72 | BAC | RMCE |
| Major vault protein | 4 | 0,0000 | 2,69 | BAC | RMCE |
| 182 kDa tankyrase-1-binding protein | 6 | 0,0000 | 2,60 | BAC | RMCE |
| V-type proton ATPase subunit B, brain isoform | 2 | 0,0000 | 2,57 | BAC | RMCE |
| Protein canopy-like 3 | 2 | 0,0000 | 2,57 | BAC | RMCE |
| Serine/threonine-protein phosphatase 2A 55 kDa regulatory subunit B alpha isoform | 3 | 0,0000 | 2,55 | BAC | RMCE |
| Annexin A11 | 5 | 0,0000 | 2,46 | BAC | RMCE |
| Dipeptidyl peptidase 2 | 2 | 0,0026 | 2,44 | BAC | RMCE |
| Receptor expression-enhancing protein 5 | 2 | 0,0000 | 2,42 | BAC | RMCE |
| Sterol-4-alpha-carboxylate 3-dehydrogenase, decarboxylating | 2 | 0,0000 | 2,38 | BAC | RMCE |
| Mannose-1-phosphate guanyltransferase beta | 2 | 0,0000 | 2,32 | BAC | RMCE |
| EH domain-containing protein 4 | 6 | 0,0010 | 2,30 | BAC | RMCE |
| Alpha-N-acetylgalactosaminidase | 4 | 0,0007 | 2,30 | BAC | RMCE |
| Peroxiredoxin-6 | 5 | 0,0000 | 2,30 | BAC | RMCE |
| Epoxide hydrolase 1 | 7 | 0,0000 | 2,30 | BAC | RMCE |
| ^5^Annexin A4 | 3 | 0,0001 | 2,27 | BAC | RMCE |
| Pyridoxal-dependent decarboxylase domain-containing protein 1 | 2 | 0,0000 | 2,27 | BAC | RMCE |
| ^2,4,6,9^Vimentin | 27 | 0,0006 | 2,26 | BAC | RMCE |
| ^10^Gelsolin | 2 | 0,0014 | 2,25 | BAC | RMCE |
| Destrin | 3 | 0,0015 | 2,17 | BAC | RMCE |
| Guanine nucleotide-binding protein G(I)/G(S)/G(O) subunit gamma-12 | 2 | 0,0000 | 2,16 | BAC | RMCE |
| Isoleucyl-tRNA synthetase, mitochondrial | 5 | 0,0000 | 2,16 | BAC | RMCE |
| Dynactin subunit 1 | 3 | 0,0002 | 2,16 | BAC | RMCE |
| ^3^Protein disulfide-isomerase A5 | 2 | 0,0016 | 2,15 | BAC | RMCE |
| Coronin-1B | 7 | 0,0000 | 2,14 | BAC | RMCE |
| General vesicular transport factor p115 | 2 | 0,0000 | 2,12 | BAC | RMCE |
| Proteasome subunit alpha type-6 | 2 | 0,0248 | 2,10 | BAC | RMCE |
| Coronin-7 | 2 | 0,0035 | 2,10 | BAC | RMCE |
| V-type proton ATPase subunit G 1 | 2 | 0,0000 | 2,10 | BAC | RMCE |
| ^4,9^Vinculin isoform 1 | 5 | 0,0156 | 2,09 | BAC | RMCE |
| Legumain | 3 | 0,0002 | 2,09 | BAC | RMCE |
| Sorbitol dehydrogenase | 5 | 0,0000 | 2,09 | BAC | RMCE |
| Neutral amino acid transporter A | 4 | 0,0001 | 2,08 | BAC | RMCE |
| Glycerol-3-phosphate dehydrogenase 1-like protein | 2 | 0,0001 | 2,07 | BAC | RMCE |
| Cathepsin D | 3 | 0,0004 | 2,06 | BAC | RMCE |
| Serine/threonine-protein phosphatase 2A 65 kDa regulatory subunit A alpha isoform | 4 | 0,0231 | 2,03 | BAC | RMCE |
| Proteasome subunit beta type-5 | 3 | 0,0000 | 2,01 | BAC | RMCE |
| Actin-related protein 2/3 complex subunit 1B | 3 | 0,0029 | 2,01 | BAC | RMCE |
| Thioredoxin domain-containing protein 12 | 2 | 0,0000 | 1,99 | BAC | RMCE |
| Transmembrane protein 43 | 3 | 0,0000 | 1,98 | BAC | RMCE |
| Alpha-2-macroglobulin receptor-associated protein | 5 | 0,0014 | 1,98 | BAC | RMCE |
| Nucleobindin-2 | 3 | 0,0000 | 1,98 | BAC | RMCE |
| Plastin-3 | 7 | 0,0000 | 1,98 | BAC | RMCE |
| Calcium-binding mitochondrial carrier protein SCaMC-1 | 3 | 0,0001 | 1,95 | BAC | RMCE |
| Ribosome-binding protein 1 | 11 | 0,0000 | 1,94 | BAC | RMCE |
| ^2,3^Protein disulfide-isomerase A3 | 24 | 0,0000 | 1,93 | BAC | RMCE |
| 3-hydroxyisobutyrate dehydrogenase, mitochondrial | 2 | 0,0000 | 1,92 | BAC | RMCE |
| ^4^Annexin A5 | 5 | 0,0002 | 1,91 | BAC | RMCE |
| ^6^Peptidyl-prolyl cis-trans isomerase B | 7 | 0,0000 | 1,90 | BAC | RMCE |
| Lysosomal alpha-glucosidase | 3 | 0,0003 | 1,87 | BAC | RMCE |
| Transgelin-2 | 3 | 0,0004 | 1,86 | BAC | RMCE |
| Pyruvate carboxylase, mitochondrial | 7 | 0,0000 | 1,85 | BAC | RMCE |
| 3-keto-steroid reductase | 2 | 0,0006 | 1,84 | BAC | RMCE |
| Estradiol 17-beta-dehydrogenase 12 | 3 | 0,0007 | 1,84 | BAC | RMCE |
| Rho GTPase-activating protein 1 | 4 | 0,0000 | 1,81 | BAC | RMCE |
| Talin-1 isoform 1 | 8 | 0,0000 | 1,81 | BAC | RMCE |
| S-formylglutathione hydrolase | 5 | 0,0000 | 1,81 | BAC | RMCE |
| Sulfated glycoprotein 1 | 12 | 0,0001 | 1,80 | BAC | RMCE |
| Alpha-actinin-1 | 16 | 0,0000 | 1,79 | BAC | RMCE |
| Sorcin | 2 | 0,0000 | 1,78 | BAC | RMCE |
| Septin-11 | 6 | 0,0000 | 1,76 | BAC | RMCE |
| V-type proton ATPase subunit E 1 | 2 | 0,0000 | 1,75 | BAC | RMCE |
| Cation-independent mannose-6-phosphate receptor | 4 | 0,0122 | 1,75 | BAC | RMCE |
| Calmodulin | 4 | 0,0008 | 1,75 | BAC | RMCE |
| Dipeptidyl-peptidase 3 | 3 | 0,0000 | 1,74 | BAC | RMCE |
| Vesicle-associated membrane protein-associated protein A | 2 | 0,0006 | 1,74 | BAC | RMCE |
| HIV Tat-specific factor 1 homolog | 4 | 0,0000 | 1,73 | BAC | RMCE |
| Mesencephalic astrocyte-derived neurotrophic factor | 5 | 0,0008 | 1,72 | BAC | RMCE |
| ^4^UDP-N-acetylhexosamine pyrophosphorylase-like protein 1 | 2 | 0,0009 | 1,72 | BAC | RMCE |
| Drebrin-like protein | 4 | 0,0000 | 1,71 | BAC | RMCE |
| Eukaryotic translation initiation factor 4 gamma 1 | 2 | 0,0077 | 1,71 | BAC | RMCE |
| ^6,9^Hypoxia up-regulated protein 1 | 5 | 0,0018 | 1,71 | BAC | RMCE |
| ^4^Adenylyl cyclase-associated protein 1 | 8 | 0,0001 | 1,70 | BAC | RMCE |
| LIM and SH3 domain protein 1 | 9 | 0,0004 | 1,69 | BAC | RMCE |
| Eukaryotic translation initiation factor 5A-1 | 2 | 0,0002 | 1,68 | BAC | RMCE |
| ^9,10^Moesin | 10 | 0,0001 | 1,68 | BAC | RMCE |
| Methylosome protein 50 | 2 | 0,0000 | 1,67 | BAC | RMCE |
| Ras-related protein Rab-1A | 3 | 0,0000 | 1,66 | BAC | RMCE |
| ^9^Cytosolic non-specific dipeptidase | 3 | 0,0005 | 1,66 | BAC | RMCE |
| Myosin-9 | 2 | 0,0022 | 1,66 | BAC | RMCE |
| Annexin A6 | 5 | 0,0008 | 1,65 | BAC | RMCE |
| E3 SUMO-protein ligase RanBP2 | 2 | 0,0041 | 1,65 | BAC | RMCE |
| Laminin subunit gamma-1 | 2 | 0,0151 | 1,65 | BAC | RMCE |
| Vesicle-trafficking protein SEC22b | 3 | 0,0006 | 1,65 | BAC | RMCE |
| Spectrin alpha chain, brain | 2 | 0,0035 | 1,64 | BAC | RMCE |
| ^2,4,7^Protein disulfide-isomerase A6 | 2 | 0,0004 | 1,64 | BAC | RMCE |
| Peptidyl-prolyl cis-trans isomerase FKBP1A | 2 | 0,0340 | 1,63 | BAC | RMCE |
| ^9^Microtubule-associated protein 1B | 6 | 0,0000 | 1,62 | BAC | RMCE |
| Sialic acid synthase | 3 | 0,0000 | 1,62 | BAC | RMCE |
| UPF0556 protein C19orf10-like | 2 | 0,0040 | 1,59 | BAC | RMCE |
| Putative RNA-binding protein Luc7-like 2 | 2 | 0,0009 | 1,59 | BAC | RMCE |
| Golgi reassembly-stacking protein 2 | 2 | 0,0017 | 1,56 | BAC | RMCE |
| Plasminogen activator inhibitor 1 RNA-binding protein | 3 | 0,0095 | 1,56 | BAC | RMCE |
| Uncharacterized protein C10orf88 homolog | 2 | 0,0433 | 1,56 | BAC | RMCE |
| Src substrate cortactin | 4 | 0,0000 | 1,56 | BAC | RMCE |
| COP9 signalosome complex subunit 1 | 2 | 0,0011 | 1,56 | BAC | RMCE |
| Kinesin-1 heavy chain | 7 | 0,0005 | 1,55 | BAC | RMCE |
| Myosin-10 | 2 | 0,0056 | 1,55 | BAC | RMCE |
| ^2,3,5,6^Annexin A1 | 11 | 0,0009 | 1,54 | BAC | RMCE |
| ^10^Hydroxymethylglutaryl-CoA synthase, cytoplasmic | 5 | 0,0090 | 1,54 | BAC | RMCE |
| Ubiquitin-conjugating enzyme E2 L3 | 2 | 0,0006 | 1,53 | BAC | RMCE |
| Protein canopy-like 2 | 3 | 0,0000 | 1,53 | BAC | RMCE |
| Catalase | 11 | 0,0001 | 1,52 | BAC | RMCE |
| ^1^Cofilin-1 | 5 | 0,0009 | 1,52 | BAC | RMCE |
| Cullin-associated NEDD8-dissociated protein 1 | 2 | 0,0039 | 1,52 | BAC | RMCE |
| ^4^Aldose reductase-related protein 2 | 4 | 0,0000 | 6,81 | RMCE | BAC |
| Heme oxygenase 1 | 7 | 0,0000 | 4,81 | RMCE | BAC |
| Agrin | 2 | 0,0077 | 3,82 | RMCE | BAC |
| Proteasome-associated protein ECM29-like | 2 | 0,0000 | 3,71 | RMCE | BAC |
| Elongation factor 1-delta | 2 | 0,0000 | 3,23 | RMCE | BAC |
| Heterogeneous nuclear ribonucleoprotein H3 | 2 | 0,0000 | 2,91 | RMCE | BAC |
| Succinate dehydrogenase [ubiquinone] iron-sulfur subunit, mitochondrial | 3 | 0,0000 | 2,87 | RMCE | BAC |
| Basement membrane-specific heparan sulfate proteoglycan core protein | 24 | 0,0000 | 2,79 | RMCE | BAC |
| Lipoprotein lipase | 2 | 0,0000 | 2,75 | RMCE | BAC |
| Succinate-semialdehyde dehydrogenase, mitochondrial | 2 | 0,0001 | 2,42 | RMCE | BAC |
| Transcription intermediary factor 1-beta | 3 | 0,0000 | 2,33 | RMCE | BAC |
| Lamina-associated polypeptide 2, isoforms alpha/zeta | 9 | 0,0000 | 2,22 | RMCE | BAC |
| C-4 methylsterol oxidase | 2 | 0,0000 | 2,16 | RMCE | BAC |
| Prostaglandin F2 receptor negative regulator | 2 | 0,0000 | 2,16 | RMCE | BAC |
| ^10^Inosine-5'-monophosphate dehydrogenase 2 | 2 | 0,0004 | 2,15 | RMCE | BAC |
| ^10^Thiosulfate sulfurtransferase | 2 | 0,0000 | 2,12 | RMCE | BAC |
| Nucleosome-binding protein 1 | 2 | 0,0048 | 2,10 | RMCE | BAC |
| Heterogeneous nuclear ribonucleoprotein A1 | 3 | 0,0000 | 2,04 | RMCE | BAC |
| 39S ribosomal protein L12, mitochondrial | 3 | 0,0000 | 2,03 | RMCE | BAC |
| Exportin-5 | 2 | 0,0001 | 1,97 | RMCE | BAC |
| DnaJ homolog subfamily A member 1 | 2 | 0,0006 | 1,94 | RMCE | BAC |
| Putative ribosomal RNA methyltransferase NOP2 | 7 | 0,0009 | 1,94 | RMCE | BAC |
| Chromobox protein homolog 3 | 4 | 0,0000 | 1,94 | RMCE | BAC |
| Cysteine desulfurase, mitochondrial | 3 | 0,0000 | 1,94 | RMCE | BAC |
| Putative adenosylhomocysteinase 2 | 3 | 0,0000 | 1,93 | RMCE | BAC |
| Mitochondrial carnitine/acylcarnitine carrier protein | 3 | 0,0004 | 1,92 | RMCE | BAC |
| Serpin H1 | 3 | 0,0006 | 1,91 | RMCE | BAC |
| Anamorsin | 2 | 0,0000 | 1,90 | RMCE | BAC |
| Succinate dehydrogenase [ubiquinone] flavoprotein subunit, mitochondrial | 13 | 0,0000 | 1,83 | RMCE | BAC |
| Proline synthase co-transcribed bacterial homolog protein | 2 | 0,0008 | 1,81 | RMCE | BAC |
| FK506-binding protein 4 | 8 | 0,0000 | 1,81 | RMCE | BAC |
| Condensin complex subunit 1 | 2 | 0,0006 | 1,81 | RMCE | BAC |
| ^7^Myb-binding protein 1A | 14 | 0,0020 | 1,80 | RMCE | BAC |
| ERO1-like protein alpha | 2 | 0,0014 | 1,79 | RMCE | BAC |
| Basic leucine zipper and W2 domain-containing protein 2 | 5 | 0,0000 | 1,78 | RMCE | BAC |
| Ubiquitin-like modifier-activating enzyme 1 | 2 | 0,0001 | 1,78 | RMCE | BAC |
| [Protein ADP-ribosylarginine] hydrolase | 2 | 0,0000 | 1,78 | RMCE | BAC |
| Nucleolar phosphoprotein p130 | 3 | 0,0218 | 1,76 | RMCE | BAC |
| Eukaryotic initiation factor 4A-II | 2 | 0,0004 | 1,75 | RMCE | BAC |
| ^4^Thymidylate synthase | 3 | 0,0133 | 1,75 | RMCE | BAC |
| Deoxyuridine 5'-triphosphate nucleotidohydrolase | 3 | 0,0124 | 1,74 | RMCE | BAC |
| ^2,5,6^60 kDa heat shock protein, mitochondrial | 18 | 0,0000 | 1,73 | RMCE | BAC |
| rRNA 2'-O-methyltransferase fibrillarin | 4 | 0,0007 | 1,73 | RMCE | BAC |
| Inorganic pyrophosphatase 2, mitochondrial | 2 | 0,0001 | 1,72 | RMCE | BAC |
| ^1^Dihydrolipoyl dehydrogenase, mitochondrial | 3 | 0,0000 | 1,72 | RMCE | BAC |
| ATPase family AAA domain-containing protein 3 | 3 | 0,0001 | 1,71 | RMCE | BAC |
| Intracellular adhesion molecule 1 | 7 | 0,0002 | 1,71 | RMCE | BAC |
| Sjogren syndrome antigen B | 2 | 0,0006 | 1,70 | RMCE | BAC |
| ^4,7^Nucleophosmin | 3 | 0,0004 | 1,70 | RMCE | BAC |
| ^4,5^Calponin-3 | 5 | 0,0032 | 1,69 | RMCE | BAC |
| NHP2-like protein 1 | 3 | 0,0002 | 1,68 | RMCE | BAC |
| High mobility group protein B2 | 2 | 0,0082 | 1,68 | RMCE | BAC |
| Lamin-B1 | 6 | 0,0000 | 1,67 | RMCE | BAC |
| ^7^DNA topoisomerase 2-alpha | 5 | 0,0004 | 1,66 | RMCE | BAC |
| Structural maintenance of chromosomes protein 4 | 2 | 0,0048 | 1,66 | RMCE | BAC |
| Histone H2B type 1-H | 2 | 0,0004 | 1,65 | RMCE | BAC |
| Ribose-phosphate pyrophosphokinase 1 | 2 | 0,0000 | 1,64 | RMCE | BAC |
| 4F2 cell-surface antigen heavy chain | 2 | 0,0002 | 1,63 | RMCE | BAC |
| Nuclear autoantigenic sperm protein | 7 | 0,0001 | 1,63 | RMCE | BAC |
| tRNA (cytosine-5-)-methyltransferase NSUN2 | 4 | 0,0000 | 1,61 | RMCE | BAC |
| ^6^Annexin A2 | 8 | 0,0132 | 1,61 | RMCE | BAC |
| Heterogeneous nuclear ribonucleoprotein L | 4 | 0,0000 | 1,60 | RMCE | BAC |
| Heterogeneous nuclear ribonucleoprotein L | 5 | 0,0000 | 1,60 | RMCE | BAC |
| Protein phosphatase 1G | 2 | 0,0009 | 1,59 | RMCE | BAC |
| Serine/arginine-rich splicing factor 7 | 4 | 0,0018 | 1,58 | RMCE | BAC |
| Torsin-1B-like | 2 | 0,0010 | 1,57 | RMCE | BAC |
| Thioredoxin domain-containing protein 5 | 4 | 0,0000 | 1,57 | RMCE | BAC |
| Dihydrolipoyllysine-residue succinyltransferase component of 2-oxoglutarate dehydrogenase complex, mitochondrial | 2 | 0,0000 | 1,56 | RMCE | BAC |
| ^6,7^High mobility group protein B1 | 5 | 0,0043 | 1,56 | RMCE | BAC |
| Polyadenylate-binding protein 4 | 3 | 0,0004 | 1,56 | RMCE | BAC |
| U3 small nucleolar RNA-interacting protein 2 | 2 | 0,0000 | 1,55 | RMCE | BAC |
| DnaJ homolog subfamily C member 7 | 2 | 0,0019 | 1,55 | RMCE | BAC |
| Heterogeneous nuclear ribonucleoprotein F | 4 | 0,0000 | 1,55 | RMCE | BAC |
| Structural maintenance of chromosomes protein 2 | 3 | 0,0036 | 1,55 | RMCE | BAC |
| ^10^RuvB-like 2 | 4 | 0,0001 | 1,53 | RMCE | BAC |
| Nucleolin | 9 | 0,0001 | 1,53 | RMCE | BAC |
| Septin-9 | 2 | 0,0004 | 1,52 | RMCE | BAC |
| ^10^ADP-sugar pyrophosphatase | 3 | 0,0000 | 1,52 | RMCE | BAC |
| ^1,7^Voltage-dependent anion-selective channel protein 1 | 6 | 0,0000 | 1,52 | RMCE | BAC |
| GTP:AMP phosphotransferase, mitochondrial | 2 | 0,0002 | 1,52 | RMCE | BAC |
| Protein RCC2 | 5 | 0,0015 | 1,51 | RMCE | BAC |
| Proliferation-associated protein 2G4 | 10 | 0,0007 | 1,50 | RMCE | BAC |
| Monocarboxylate transporter 1 | 3 | 0,0000 | 1,50 | RMCE | BAC |

^1-10^ also mentioned in: ^1^Alete et al., 2005; ^2^Baik et al., 2006; ^3^Baik et al., 2008; ^4^Meleady et al., 2008; ^5^Meleady et al., 2011; ^6^Nissom et al., 2006; ^7^Seth et al., 2007; ^8^Van Dyk et al., 2003; ^9^Wingens et al., 2015; ^10^Yee et al., 2008

**Supplemental Table 4:** Transgene delivery comparison RMCE (n=6) vs Plasmid (n=6) vs BAC (n=6); same expression pattern for 2F5-scFv-Fc and 3D6-scFv-Fc; Combined identified differential proteins (n=58+1); number of peptides used for quantitation ≥ 2; Anova p Value ≤ 0.05; fold change **≥ 1.5x** in both in-group comparisons. Table is sorted by fold-change and positive or negative correlation with qP.

|  |  | **3D6** | | | **2F5** | | |  |  |
| --- | --- | --- | --- | --- | --- | --- | --- | --- | --- |
| **Description** | **Gene ID** | **Peptides used for quant.** | **Anova (p)** | **Fold change** | **Peptides used for quant.** | **Anova (p)** | **fold change** | **Highest mean** | **Lowest mean** |
| Leukocyte elastase inhibitor A | Serpinb1a | 8 | 0.000 | **18.04** | 7 | 0.000 | **5.77** | BAC | RMCE |
| ***Ig gamma-1 chain C region*** | ***IGHG1*** | 7 | 0.000 | ***10.13*** | 11 | 0.000 | ***4.62*** | BAC | RMCE |
| Cathepsin B | Ctsb | 6 | 0.000 | **5.14** | 7 | 0.000 | **3.51** | BAC | RMCE |
| Galectin-1 | Lgals1 | 8 | 0.000 | **4.81** | 9 | 0.000 | **4.91** | BAC | RMCE |
| Myoferlin | Myof | 2 | 0.000 | **4.58** | 2 | 0.000 | **2.28** | BAC | RMCE |
| Macrophage-capping protein | Capg | 5 | 0.000 | **4.17** | 5 | 0.000 | **2.27** | BAC | RMCE |
| Cysteine-rich with EGF-like domain protein 2 | Creld2 | 3 | 0.000 | **4.16** | 3 | 0.000 | **3.01** | BAC | RMCE |
| NADH-cytochrome b5 reductase 1 | Cyb5r1 | 2 | 0.000 | **3.65** | 2 | 0.000 | **3.76** | BAC | RMCE |
| LIM domain and actin-binding protein 1 | Lima1 | 5 | 0.000 | **2.83** | 4 | 0.003 | **2.10** | BAC | RMCE |
| Plectin | Plec | 3 | 0.000 | **2.74** | 2 | 0.000 | **2.77** | BAC | RMCE |
| V-type proton ATPase subunit B, brain isoform | Atp6v1b2 | 2 | 0.000 | **2.57** | 2 | 0.001 | **1.58** | BAC | RMCE |
| Serine/threonine-protein phosphatase 2A 55 kDa regulatory subunit B alpha isoform | Ppp2r2a | 3 | 0.000 | **2.55** | 3 | 0.000 | **1.89** | BAC | RMCE |
| Annexin A11 | Anxa11 | 5 | 0.000 | **2.46** | 5 | 0.001 | **1.66** | BAC | RMCE |
| Receptor expression-enhancing protein 5 | Reep5 | 2 | 0.000 | **2.42** | 2 | 0.000 | **1.66** | BAC | RMCE |
| Alpha-N-acetylgalactosaminidase | Naga | 4 | 0.001 | **2.30** | 4 | 0.000 | **3.20** | BAC | RMCE |
| Pyridoxal-dependent decarboxylase domain-containing protein 1 | Pdxdc1 | 2 | 0.000 | **2.27** | 2 | 0.000 | **2.11** | BAC | RMCE |
| Vimentin | Vim | 27 | 0.001 | **2.26** | 24 | 0.002 | **2.31** | BAC | RMCE |
| Gelsolin | Gsn | 2 | 0.001 | **2.25** | 3 | 0.000 | **2.57** | BAC | RMCE |
| Destrin | Dstn | 3 | 0.001 | **2.17** | 3 | 0.001 | **1.75** | BAC | RMCE |
| Guanine nucleotide-binding protein G(I)/G(S)/G(O) subunit gamma-12 | Gng12 | 2 | 0.000 | **2.16** | 2 | 0.000 | **1.70** | BAC | RMCE |
| Isoleucyl-tRNA synthetase, mitochondrial | Iars2 | 5 | 0.000 | **2.16** | 5 | 0.000 | **2.41** | BAC | RMCE |
| Coronin-1B | Coro1b | 7 | 0.000 | **2.14** | 8 | 0.000 | **2.08** | BAC | RMCE |
| V-type proton ATPase subunit G 1 | Atp6v1g1 | 2 | 0.000 | **2.10** | 2 | 0.001 | **1.58** | BAC | RMCE |
| Glycerol-3-phosphate dehydrogenase 1-like protein | Gpd1l | 2 | 0.000 | **2.07** | 2 | 0.002 | **2.17** | BAC | RMCE |
| Actin-related protein 2/3 complex subunit 1B | Arpc1b | 3 | 0.003 | **2.01** | 2 | 0.006 | **2.71** | BAC | RMCE |
| Transmembrane protein 43 | Tmem43 | 3 | 0.000 | **1.98** | 2 | 0.000 | **1.96** | BAC | RMCE |
| Nucleobindin-2 | Nucb2 | 3 | 0.000 | **1.98** | 3 | 0.000 | **1.85** | BAC | RMCE |
| Peptidyl-prolyl cis-trans isomerase B | Ppib | 7 | 0.000 | **1.90** | 7 | 0.000 | **1.94** | BAC | RMCE |
| Transgelin-2 | Tagln2 | 3 | 0.000 | **1.86** | 4 | 0.000 | **2.15** | BAC | RMCE |
| Pyruvate carboxylase, mitochondrial | Pcx | 7 | 0.000 | **1.85** | 2 | 0.014 | **1.95** | BAC | RMCE |
| 3-keto-steroid reductase | Hsd17b7 | 2 | 0.001 | **1.84** | 2 | 0.000 | **1.61** | BAC | RMCE |
| Estradiol 17-beta-dehydrogenase 12 | Hsd17b12 | 3 | 0.001 | **1.84** | 3 | 0.000 | **1.85** | BAC | RMCE |
| Rho GTPase-activating protein 1 | Arhgap1 | 4 | 0.000 | **1.81** | 4 | 0.000 | **2.05** | BAC | RMCE |
| Sulfated glycoprotein 1 | Psap | 12 | 0.000 | **1.80** | 10 | 0.000 | **1.84** | BAC | RMCE |
| Septin-11 | Sept11 | 6 | 0.000 | **1.76** | 2 | 0.001 | **1.57** | BAC | RMCE |
| Calmodulin | Calm1 | 4 | 0.001 | **1.75** | 3 | 0.001 | **1.62** | BAC | RMCE |
| Vesicle-associated membrane protein-associated protein A | Vapa | 2 | 0.001 | **1.74** | 5 | 0.011 | **1.56** | BAC | RMCE |
| HIV Tat-specific factor 1 homolog | Htatsf1 | 4 | 0.000 | **1.73** | 2 | 0.012 | **1.97** | BAC | RMCE |
| LIM and SH3 domain protein 1 | Lasp1 | 9 | 0.000 | **1.69** | 8 | 0.000 | **1.66** | BAC | RMCE |
| Ras-related protein Rab-1A | Rab1 | 3 | 0.000 | **1.66** | 2 | 0.000 | **2.01** | BAC | RMCE |
| Myosin-9 | Myh9 | 2 | 0.002 | **1.66** | 2 | 0.004 | **2.79** | BAC | RMCE |
| Vesicle-trafficking protein SEC22b | Sec22b | 3 | 0.001 | **1.65** | 3 | 0.003 | **1.69** | BAC | RMCE |
| Microtubule-associated protein 1B | Mtap1b | 6 | 0.000 | **1.62** | 2 | 0.000 | **1.89** | BAC | RMCE |
| Src substrate cortactin | Cttn | 4 | 0.000 | **1.56** | 6 | 0.002 | **1.76** | BAC | RMCE |
| Myosin-10 | Myh10 | 2 | 0.006 | **1.55** | 3 | 0.001 | **2.05** | BAC | RMCE |
| Protein canopy-like 2 | Cnpy2 | 3 | 0.000 | **1.53** | 3 | 0.000 | **1.56** | BAC | RMCE |
|  |  |  |  |  |  |  |  |  |  |
| Heme oxygenase 1 | Hmox1 | 7 | 0.000 | **4.81** | 7 | 0.000 | **3.41** | RMCE | BAC |
| Proteasome-associated protein ECM29-like | Ecm29 | 2 | 0.000 | **3.71** | 2 | 0.003 | **2.21** | RMCE | BAC |
| Lipoprotein lipase | Lpl | 2 | 0.000 | **2.75** | 2 | 0.000 | **2.99** | RMCE | BAC |
| Lamina-associated polypeptide 2, isoforms alpha/zeta | Tmpo | 9 | 0.000 | **2.22** | 8 | 0.000 | **1.89** | RMCE | BAC |
| Nucleosome-binding protein 1 | Hmgn5 | 2 | 0.005 | **2.10** | 3 | 0.006 | **2.80** | RMCE | BAC |
| Exportin-5 | Xpo5 | 2 | 0.000 | **1.97** | 2 | 0.000 | **2.33** | RMCE | BAC |
| Anamorsin | Ciapin1 | 2 | 0.000 | **1.90** | 3 | 0.000 | **2.31** | RMCE | BAC |
| Basic leucine zipper and W2 domain-containing protein 2 | Bzw2 | 5 | 0.000 | **1.78** | 5 | 0.000 | **1.68** | RMCE | BAC |
| Intracellular adhesion molecule 1 | Icam1 | 7 | 0.000 | **1.71** | 8 | 0.000 | **2.30** | RMCE | BAC |
| Ribose-phosphate pyrophosphokinase 1 | Prps1 | 2 | 0.000 | **1.64** | 3 | 0.000 | **1.87** | RMCE | BAC |
| Nuclear autoantigenic sperm protein | Nasp | 7 | 0.000 | **1.63** | 6 | 0.000 | **1.61** | RMCE | BAC |
| Protein phosphatase 1G | Ppm1g | 2 | 0.001 | **1.59** | 3 | 0.000 | **1.91** | RMCE | BAC |
| Proliferation-associated protein 2G4 | Pa2g4 | 10 | 0.001 | **1.50** | 10 | 0.000 | **1.59** | RMCE | BAC |

**Supplemental Table 5**: Transgene delivery comparison RMCE (n=6) vs Plasmid (n=6) vs BAC (n=6); 2F5-scFv-Fc identified differential proteins (n=9) correlating to qP or µ that were not identified in the in-group comparison of 3D6-scFv-Fc at all; number of peptides used for quantitation ≥ 2; Anova p Value ≤ 0.05; fold change **≥ 1.5x** in both in-group comparisons. Table is sorted by fold-change and positive or negative correlation with qP.

| **Description** | **Gene ID** | **Fold change** | **Highest mean** | **Lowest mean** |
| --- | --- | --- | --- | --- |
| Heat shock protein beta-1 | Hspb1 | **2.51** | BAC | RMCE |
| Translocation protein SEC62 | Sec62 | **2.01** | BAC | RMCE |
| MHC class I antigen Hm1-C4 | - | **1.66** | BAC | RMCE |
| Dolichyl-diphosphooligosaccharide--protein glycosyltransferase subunit STT3A | Stt3a | **1.57** | BAC | RMCE |
| Thioredoxin reductase 1, cytoplasmic | Txnrd1 | **1.97** | RMCE | BAC |
| Proliferating cell nuclear antigen | Pcna | **1.71** | RMCE | BAC |
| Cullin-4B | Cul4b | **1.63** | RMCE | BAC |
| Glutathione S-transferase P 2 | Gstp2 | **1.63** | RMCE | BAC |
| Eukaryotic translation initiation factor 3 subunit J | Eif3j | **1.57** | RMCE | BAC |

**Supplemental Table 6:** Transgene delivery comparison RMCE (n=6) vs Plasmid (n=6) vs BAC (n=6); 3D6 scFv-Fc identified differential proteins (n=32) correlating to qP or µ that were not identified in the in-group comparison of 2F5-scFv-Fc at all; number of peptides used for quantitation ≥ 2; Anova p Value ≤ 0.05; fold change **≥ 1.5x** in both in-group comparisons. Table is sorted by fold-change and positive or negative correlation with qP.

| **Description** | **Gene ID** | **Fold change** | **Highest mean** | **Lowest mean** |
| --- | --- | --- | --- | --- |
| Epididymal secretory protein E1 | Npc2 | **2.9** | **BAC** | **RMCE** |
| 60S acidic ribosomal protein P2 | Rplp2 | **2.74** | **BAC** | **RMCE** |
| Sterol-4-alpha-carboxylate 3-dehydrogenase, decarboxylating | Nsdhl | **2.38** | **BAC** | **RMCE** |
| Mannose-1-phosphate guanyltransferase beta | Gmppb | **2.32** | **BAC** | **RMCE** |
| Dynactin subunit 1 | Dctn1 | **2.16** | **BAC** | **RMCE** |
| Proteasome subunit alpha type-6 | Psma6 | **2.1** | **BAC** | **RMCE** |
| Coronin-7 | Coro7 | **2.1** | **BAC** | **RMCE** |
| Vinculin isoform 1 | Vcl | **2.09** | **BAC** | **RMCE** |
| Legumain | Lgmn | **2.09** | **BAC** | **RMCE** |
| Serine/threonine-protein phosphatase 2A 65 kDa regulatory subunit A alpha isoform | Ppp2r1a | **2.03** | **BAC** | **RMCE** |
| Proteasome subunit beta type-5 | Psmb5 | **2.01** | **BAC** | **RMCE** |
| Cation-independent mannose-6-phosphate receptor | Igf2r | **1.75** | **BAC** | **RMCE** |
| Eukaryotic translation initiation factor 4 gamma 1 | Eif4g1 | **1.71** | **BAC** | **RMCE** |
| Peptidyl-prolyl cis-trans isomerase FKBP1A | Fkbp1a | **1.63** | **BAC** | **RMCE** |
| Putative RNA-binding protein Luc7-like 2 | Luc7l2 | **1.59** | **BAC** | **RMCE** |
| Golgi reassembly-stacking protein 2 | Gorasp2 | **1.56** | **BAC** | **RMCE** |
| Plasminogen activator inhibitor 1 RNA-binding protein | Serbp1 | **1.56** | **BAC** | **RMCE** |
| Hydroxymethylglutaryl-CoA synthase, cytoplasmic | Hmgcs1 | **1.54** | **BAC** | **RMCE** |
| Ubiquitin-conjugating enzyme E2 L3 | Ube2l3 | **1.53** | **BAC** | **RMCE** |
| Agrin | Agrn | **3.82** | **RMCE** | **BAC** |
| Succinate-semialdehyde dehydrogenase, mitochondrial | Aldh5a1 | **2.42** | **RMCE** | **BAC** |
| C-4 methylsterol oxidase | Sc4mol | **2.16** | **RMCE** | **BAC** |
| Thiosulfate sulfurtransferase | Tst | **2.12** | **RMCE** | **BAC** |
| Mitochondrial carnitine/acylcarnitine carrier protein | Slc25a20 | **1.92** | **RMCE** | **BAC** |
| Thymidylate synthase | Tyms | **1.75** | **RMCE** | **BAC** |
| Structural maintenance of chromosomes protein 4 | Smc4 | **1.66** | **RMCE** | **BAC** |
| Histone H2B type 1-H | Hist1h2bh | **1.65** | **RMCE** | **BAC** |
| tRNA (cytosine-5-)-methyltransferase NSUN2 | Nsun2 | **1.61** | **RMCE** | **BAC** |
| Thioredoxin domain-containing protein 5 | Txndc5 | **1.57** | **RMCE** | **BAC** |
| High mobility group protein B1 | Hmgb1 | **1.56** | **RMCE** | **BAC** |
| Nucleolin | Ncl | **1.53** | **RMC E** | **BAC** |
| Septin-9 | Sept9 | **1.52** | **RMCE** | **BAC** |

**Supplemental Table 7:** Transgene delivery comparison RMCE (n=6) vs Plasmid (n=6) vs BAC (n=6); differential expressed proteins correlating with qP or µ showing the opposite expression pattern for 3D6-scFv-Fc and 2F5-scFv-Fc producers (n=2) (number of peptides used for quantitation ≥ 2; Anova p Value ≤ 0.05; fold change **≥ 1.5x** in at least one of the two in-group comparisons).

|  |  | **3D6** | | | | | **2F5** | | | | |
| --- | --- | --- | --- | --- | --- | --- | --- | --- | --- | --- | --- |
| **Description** | **Gene ID** | **Peptides used for quant.** | **Anova (p)** | **Max fold change** | **max** | **min** | **Peptides used for quant.** | **Anova (p)** | **Max fold change** | **max** | **min** |
| Cullin-associated NEDD8-dissociated protein 1 | Cand1 | **2** | **0.004** | **1.52** | BAC | RMCE | 3 | 0.019 | 1.22 | RMCE | BAC |
| Protein SON | Son | **3** | **0.036** | **1.27** | BAC | RMCE | 2 | 0.001 | 1.79 | RMCE | BAC |

**Gene Enrichment Analysis**

**Supplemental Table 8:** GeneCodis3 output: GOSlim process singular enrichment analysis of gene list (n=60) of proteins identified to be differential between 2F5- and 3D6-scFv-Fc clones (transgene comparison).

| **Items** | **Details** | **Support** | **List size** | **Reference support** | **Reference size** | **hyperg. pValue** | **Genes** |
| --- | --- | --- | --- | --- | --- | --- | --- |
| GO:0008283 | cell proliferation (BP) | 3 | 60 | 159 | 37681 | 0.002 | Mki67,Prdx1,Txnrd1 |
| GO:0006457 | protein folding (BP) | 6 | 60 | 117 | 37681 | 0.000 | Grpel1,Hspe1,Hspd1,Hsp90b1, Calr,Dnajc7 |
| GO:0030198 | extracellular matrix organization (BP) | 2 | 60 | 85 | 37681 | 0.008 | Lgals3,Hspg2 |

**Supplemental Table 9:** GeneCodis3 output: GOSlim process singular enrichment of gene list (n=58) of proteins that showed the same expression profile in both transgene delivery comparisons. The protein Ecm29 was not identified by GeneCodis3 and is therefore not part of this enrichment analysis.

| **Items** | **Details** | **Support** | **List size** | **Reference support** | **Reference size** | **hyperg. pValue** | **Genes** |
| --- | --- | --- | --- | --- | --- | --- | --- |
| GO:0007049 | cell cycle (BP) | 5 | 57 | 528 | 37681 | 0.001 | Ppm1g,Calm1,Nasp, Sept11,Anxa11 |
| GO:0016192 | vesicle-mediated transport (BP) | 3 | 57 | 168 | 37681 | 0.002 | Gsn,Rab1,Sec22b |
| GO:0008219 | cell death (BP) | 2 | 57 | 50 | 37681 | 0.003 | Vapa,Hmox1 |
| GO:0006810 | transport (BP) | 7 | 57 | 1615 | 37681 | 0.011 | Xpo5,Lasp1,Atp6v1g1,Nasp, Atp6v1b2,Rab1,Sec22b |

**References**

Abdelmohsen K, Tominaga K, Lee EK, Srikantan S, Kang MJ, Kim MM, Selimyan R, Martindale JL, Yang X, Carrier F and others. 2011. Enhanced translation by Nucleolin via G-rich elements in coding and non-coding regions of target mRNAs. Nucleic Acids Res 39(19):8513-30.

Alete DE, Racher AJ, Birch JR, Stansfield SH, James DC, Smales CM. 2005. Proteomic analysis of enriched microsomal fractions from GS-NS0 murine myeloma cells with varying secreted recombinant monoclonal antibody productivities. Proteomics 5(18):4689-704.

Angelov D, Bondarenko VA, Almagro S, Menoni H, Mongelard F, Hans F, Mietton F, Studitsky VM, Hamiche A, Dimitrov S. 2006. Nucleolin is a histone chaperone with FACT‐like activity and assists remodeling of nucleosomes. The EMBO Journal 25(8):1669-1679.

Baake M, Bäuerle M, Doenecke D, Albig W. 2001. Core histones and linker histones are imported into the nucleus by different pathways. European Journal of Cell Biology 80(11):669-677.

Baik JY, Joo EJ, Kim YH, Lee GM. 2008. Limitations to the comparative proteomic analysis of thrombopoietin producing Chinese hamster ovary cells treated with sodium butyrate. J Biotechnol 133(4):461-8.

Baik JY, Lee MS, An SR, Yoon SK, Joo EJ, Kim YH, Park HW, Lee GM. 2006. Initial transcriptome and proteome analyses of low culture temperature-induced expression in CHO cells producing erythropoietin. Biotechnol Bioeng 93(2):361-71.

Dumic J, Dabelic S, Flögel M. 2006. Galectin-3: an open-ended story. Biochimica et Biophysica Acta (BBA)-General Subjects 1760(4):616-635.

Ginisty H, Amalric F, Bouvet P. 1998. Nucleolin functions in the first step of ribosomal RNA processing. The EMBO Journal 17(5):1476-1486.

Haudek KC, Spronk KJ, Voss PG, Patterson RJ, Wang JL, Arnoys EJ. 2010. Dynamics of galectin-3 in the nucleus and cytoplasm. Biochimica et Biophysica Acta (BBA)-General Subjects 1800(2):181-189.

Jäkel S, Görlich D. 1998. Importin beta, transportin, RanBP5 and RanBP7 mediate nuclear import of ribosomal proteins in mammalian cells. EMBO J 17(15):4491-502.

Kirkman HN, Gaetani GF. 2007. Mammalian catalase: a venerable enzyme with new mysteries. Trends in Biochemical Sciences 32(1):44-50.

Liu H, Ling Y, Gong Y, Sun Y, Hou L, Zhang B. 2007. DNA damage induces N-acetyltransferase NAT10 gene expression through transcriptional activation. Molecular and Cellular Biochemistry 300(1-2):249-258.

Lv J, Liu H, Wang Q, Tang Z, Hou L, Zhang B. 2003. Molecular cloning of a novel human gene encoding histone acetyltransferase-like protein involved in transcriptional activation of hTERT. Biochemical and Biophysical Research Communications 311(2):506-513.

Malhotra JD, Kaufman RJ. 2007. The endoplasmic reticulum and the unfolded protein response. Seminars in Cell & Developmental Biology 18(6):716-731.

Mebratu Y, Tesfaigzi Y. 2009. How ERK1/2 activation controls cell proliferation and cell death: Is subcellular localization the answer? Cell Cycle 8(8):1168-75.

Meleady P, Doolan P, Henry M, Barron N, Keenan J, O'Sullivan F, Clarke C, Gammell P, Melville MW, Leonard M and others. 2011. Sustained productivity in recombinant Chinese hamster ovary (CHO) cell lines: proteome analysis of the molecular basis for a process-related phenotype. BMC Biotechnol 11:78.

Meleady P, Henry M, Gammell P, Doolan P, Sinacore M, Melville M, Francullo L, Leonard M, Charlebois T, Clynes M. 2008. Proteomic profiling of CHO cells with enhanced rhBMP-2 productivity following co-expression of PACEsol. Proteomics 8(13):2611-24.

Nissom PM, Sanny A, Kok YJ, Hiang YT, Chuah SH, Shing TK, Lee YY, Wong KT, Hu WS, Sim MY and others. 2006. Transcriptome and proteome profiling to understanding the biology of high productivity CHO cells. Mol Biotechnol 34(2):125-40.

Ong YS, Tang BL, Loo LS, Hong W. 2010. p125A exists as part of the mammalian Sec13/Sec31 COPII subcomplex to facilitate ER-Golgi transport. J Cell Biol 190(3):331-45.

Rhee SG, Chang T-S, Bae YS, Lee S-R, Kang SW. 2003. Cellular Regulation by Hydrogen Peroxide. Journal of the American Society of Nephrology 14(suppl 3):S211-S215.

Rossi MR, Somji S, Garrett SH, Sens MA, Nath J, Sens DA. 2002. Expression of hsp 27, hsp 60, hsc 70, and hsp 70 stress response genes in cultured human urothelial cells (UROtsa) exposed to lethal and sublethal concentrations of sodium arsenite. Environmental health perspectives 110(12):1225.

Seth G, Philp RJ, Lau A, Jiun KY, Yap M, Hu WS. 2007. Molecular portrait of high productivity in recombinant NS0 cells. Biotechnol Bioeng 97(4):933-51.

Shimoi W, Ezawa I, Nakamoto K, Uesaki S, Gabreski G, Aridor M, Yamamoto A, Nagahama M, Tagaya M, Tani K. 2005. p125 is localized in endoplasmic reticulum exit sites and involved in their organization. J Biol Chem 280(11):10141-8.

Smeenk G, Wiegant WW, Vrolijk H, Solari AP, Pastink A, van Attikum H. 2010. The NuRD chromatin–remodeling complex regulates signaling and repair of DNA damage. The Journal of Cell Biology 190(5):741-749.

Snyder M, He W, Zhang JJ. 2005. The DNA replication factor MCM5 is essential for Stat1-mediated transcriptional activation. Proceedings of the National Academy of Sciences of the United States of America 102(41):14539-14544.

Tew KD, Manevich Y, Grek C, Xiong Y, Uys J, Townsend DM. 2011. The role of glutathione S-transferase P in signaling pathways and S-glutathionylation in cancer. Free Radical Biology and Medicine 51(2):299-313.

Van Dyk DD, Misztal DR, Wilkins MR, Mackintosh JA, Poljak A, Varnai JC, Teber E, Walsh BJ, Gray PP. 2003. Identification of cellular changes associated with increased production of human growth hormone in a recombinant Chinese hamster ovary cell line. Proteomics 3(2):147-56.

Wingens M, Gätgens J, Schmidt A, Albaum SP, Büntemeyer H, Noll T, Hoffrogge R. 2015. 2D-DIGE screening of high-productive CHO cells under glucose limitation--basic changes in the proteome equipment and hints for epigenetic effects. J Biotechnol 201:86-97.

Xue Y, Wong J, Moreno GT, Young MK, Côté J, Wang W. 1998. NURD, a Novel Complex with Both ATP-Dependent Chromatin-Remodeling and Histone Deacetylase Activities. Molecular Cell 2(6):851-861.

Yee JC, de Leon Gatti M, Philp RJ, Yap M, Hu WS. 2008. Genomic and proteomic exploration of CHO and hybridoma cells under sodium butyrate treatment. Biotechnol Bioeng 99(5):1186-204.
